# Exome capture from saliva produces high quality genomic and metagenomic data

**DOI:** 10.1186/1471-2164-15-262

**Published:** 2014-04-04

**Authors:** Jeffrey M Kidd, Thomas J Sharpton, Dean Bobo, Paul J Norman, Alicia R Martin, Meredith L Carpenter, Martin Sikora, Christopher R Gignoux, Neda Nemat-Gorgani, Alexandra Adams, Moraima Guadalupe, Xiaosen Guo, Qiang Feng, Yingrui Li, Xiao Liu, Peter Parham, Eileen G Hoal, Marcus W Feldman, Katherine S Pollard, Jeffrey D Wall, Carlos D Bustamante, Brenna M Henn

**Affiliations:** 1 Department of Genetics, Stanford University, Stanford, CA 94305, USA; 2 Departments of Human Genetics, and Computational Medicine and Bioinformatics, University of Michigan, Ann Arbor, MI, USA; 3 The J. David Gladstone Institutes, University of California, San Francisco, San Francisco, CA 94158, USA; 4 Departments of Microbiology, and Statistics, Oregon State University, Corvallis, OR 97331, USA; 5 Department of Ecology and Evolution, Stony Brook University, Life Sciences Bldg, Room 640, Stony Brook, NY 11794, USA; 6 Department of Structural Biology, Stanford University, Stanford, CA 94305, USA; 7 Program in Pharmaceutical Sciences and Pharmacogenomics, University of California, San Francisco, CA 94143, USA; 8 Agilent Technologies, Genomics Division, Cedar Creek, TX 78612, USA; 9 Translational Medicine, BGI – Shenzhen, Shenzhen, China; 10 Stellenbosch University, Tygerberg, South Africa; 11 Department of Biological Sciences, Stanford University, Stanford, CA 94305, USA; 12 Institute for Human Genetics, and the Departments of Epidemiology and Biostatistics, University of California, San Francisco, San Francisco, CA 94143, USA

**Keywords:** Exomes, KhoeSan, Genetic diversity, Metagenomics, Microbiome

## Abstract

**Background:**

Targeted capture of genomic regions reduces sequencing cost while generating higher coverage by allowing biomedical researchers to focus on specific loci of interest, such as exons. Targeted capture also has the potential to facilitate the generation of genomic data from DNA collected via saliva or buccal cells. DNA samples derived from these cell types tend to have a lower human DNA yield, may be degraded from age and/or have contamination from bacteria or other ambient oral microbiota. However, thousands of samples have been previously collected from these cell types, and saliva collection has the advantage that it is a non-invasive and appropriate for a wide variety of research.

**Results:**

We demonstrate successful enrichment and sequencing of 15 South African KhoeSan exomes and 2 full genomes with samples initially derived from saliva. The expanded exome dataset enables us to characterize genetic diversity free from ascertainment bias for multiple KhoeSan populations, including new exome data from six HGDP Namibian San, revealing substantial population structure across the Kalahari Desert region. Additionally, we discover and independently verify thirty-one previously unknown *KIR* alleles using methods we developed to accurately map and call the highly polymorphic *HLA* and *KIR* loci from exome capture data. Finally, we show that exome capture of saliva-derived DNA yields sufficient non-human sequences to characterize oral microbial communities, including detection of bacteria linked to oral disease (e.g. *Prevotella melaninogenica*). For comparison, two samples were sequenced using standard full genome library preparation without exome capture and we found no systematic bias of metagenomic information between exome-captured and non-captured data.

**Conclusions:**

DNA from human saliva samples, collected and extracted using standard procedures, can be used to successfully sequence high quality human exomes, and metagenomic data can be derived from non-human reads. We find that individuals from the Kalahari carry a higher oral pathogenic microbial load than samples surveyed in the Human Microbiome Project. Additionally, rare variants present in the exomes suggest strong population structure across different KhoeSan populations.

## Background

Sampling of saliva or via buccal cell extractions is a widely employed, non-invasive method of collecting human DNA for both biomedical and ancestry experiments. DNA extracted from saliva fluid has been used on single nucleotide polymorphism chip arrays, methylation arrays, targeted resequencing, exome, and whole genome sequencing [1-7]. However, the low total yield of DNA from a single sample and the presence of many non-human DNA fragments make next-generation sequencing of saliva samples impractical for some applications. Targeted enrichment strategies, such as hybridization methods designed to capture the exons of annotated genes (the ‘exome’) prior to sequencing, offer a way to circumvent some of the limitations posed by saliva-derived DNA samples. We demonstrate the successful sequencing of multiple human exomes from saliva-derived samples using commercially available reagents for exome capture.

Exome sequencing and other capture methods permit the high-coverage sequencing of a small portion of the genome. This approach represents a trade off between depth of coverage vs. breadth of the genome that is interrogated, and has the potential to revolutionize genomic medicine [8,9]. In addition to direct applications to human disease, exome sequencing of a modest number of individuals can reveal important aspects of human evolution [10-12]. The capability to apply these approaches to DNA derived from saliva, which is more easily obtained and less invasive than blood or other tissue collection, will greatly facilitate the detailed examination of genetic variants that may be associated with specific traits or have experienced adaptive evolution [13,14].

We focus on a unique set of DNA samples from the ≠Khomani KhoeSan of South Africa to illustrate the utility of exome sequencing via saliva. African genetic diversity remains poorly understood, in part because many regions of the continent lack adequate healthcare infrastructure, which can make blood collection impractical. The indigenous KhoeSan peoples of southern Africa are a collection of hunter-gatherer and pastoralist groups who speak “click languages”, classified into three distinct language families. The genetic diversity of these, and related populations, remains under-ascertained. The genome of one Tuu-speaking San (“!Gubi”) has been fully sequenced and found to contain over 700,000 novel polymorphisms [15]. Gronau et al. showed that this San genome was highly divergent among known genomes, even compared to other African individuals [16]. They estimated the population divergence between western African individuals and the San to be about 110,000-130,000 years ago, over twice as old as the divergence between western Africans and Eurasians. Additionally, single nucleotide polymorphism (SNP) array data demonstrated that the ≠Khomani San population had the lowest levels of linkage disequilbrium (LD) of any population surveyed and thus the largest effective population size [2]. However, in order to test hypotheses regarding population sub-structure, natural selection and biomedically relevant variants in Africa, it is essential to have both large sample sizes and genomic data that are un-biased with regard to ascertainment schemes.

## Results

Fifteen human saliva samples were selected for exome sequencing. Samples were split into two batches (“Pilot 1” and “Pilot 2”), representing samples enriched using the Agilent SureSelect 50 Mb human All-Exon design and sequenced with the Illumina GAII machine and a replication batch enriched using the Agilent SureSelect 44 Mb human All-Exon design and sequenced using Illumina HiSeq. We included a familial quartet with two daughters (Family 1), an extended pedigree of first cousins and half-siblings (Family 2), and eight purportedly unrelated individuals (Additional file 1: Figure S1). Family 1 displayed complex ancestry from KhoeSan, European and both eastern and western African populations (see [2]). Family 2 and the un-related individuals self-reported their ancestry as being from only KhoeSan populations (Nama- or N|u-speakers). We obtained 3-25ug total DNA from each saliva sample. Each aliquot was processed using the Agilent SureSelectXT library preparation kit followed by enrichment with the SureSelect 44 Mb or SureSelect 50 Mb human All-Exon capture probes. Using standard Illumina post capture barcodes, libraries were sequenced on either an Illumina GAII or HiSeq machine. Aliquots from two samples (SA1000 and SA1025) were also sequenced without exome capture, using the Illumina TruSeq library preparation kit (SA1000) and the Illumina Nextera library preparation kit (SA1025). The whole genome sequence (WGS) libraries were then sequenced on two lanes of an Illumina HiSeq.

### Sequencing statistics

**Figure 1:**
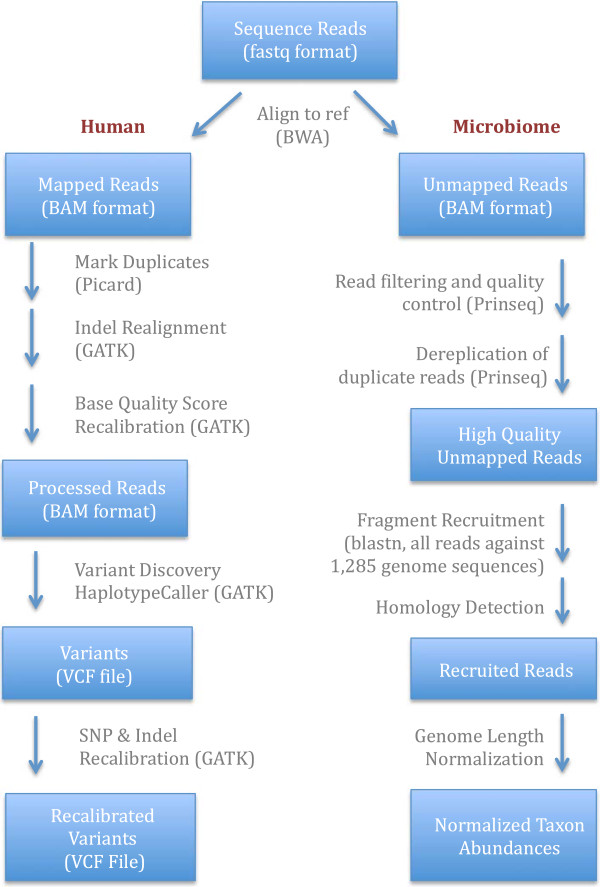
**Schematic of mapping and calling pipelines.** Each box summarizes the data and data format used for each step of the human exome and microbiome mapping/calling pipelines. The pipeline begins with next-generation sequencing raw reads obtained from exome sequencing of saliva-derived DNA and ends in finalized exome variant calls and microbiome taxonomic abundances. Arrows indicate analysis methods used to process the human and saliva microbiome data (see Methods).

An average of 76.5 million 75 bp paired reads and 84.3 million 100 bp paired reads were obtained for each individual in the Pilot 1 GAII and Pilot 2 HiSeq exome experiments (Table 1). Across all samples, 86.8%-98.1% of the reads mapped to the human genome reference (GRCh37) (Figure 1). On average, ~70-75% of non-duplicate, mapped reads fell in the specified target regions. This on-target percentage is similar to previous on-target percentages (70-87%) for standard blood or cell line-derived human DNA with Agilent SureSelect exon designs [17,18].

**Table 1:** Summary statistics for KhoeSan exomes

		**Total reads**^**a**^	**Unmapped reads**	**% Un-mapped reads**	**% PCR duplicates**	**% Mapped on target**	**Median target coverage**^**b**^	**% of variants covered**^**c**^	**Autosomal SNV**	**Autosomal singletons**	**Non-ref. concordance**^**d**^
Pilot 1	SA006	69,272,282	9,122,731	13.2%	54.2%	63.5%	12	94.9%	25,225	657	0.9897
	SA008	113,888,276	2,143,408	1.9%	19.8%	78.2%	73	99.5%	26,408	955	0.9947
	SA011	78,006,472	1,664,959	2.1%	33.7%	77.4%	40	99.0%	26,365	67	NA
	SA012	67,209,032	1,353,187	2.0%	20.5%	75.7%	42	99.3%	26,722	86	NA
	SA035	85,142,498	5,812,851	6.8%	78.0%	79.4%	10	92.1%	24,692	1,726	0.9884
	SA051	76,076,464	3,102,819	4.1%	27.8%	76.5%	37	98.8%	27,674	1,239	NA
	SA052	60,375,472	1,247,951	2.1%	12.9%	78.2%	41	98.8%	27,779	755	0.9968
	SA054	62,358,148	1,959,032	3.1%	27.9%	73.9%	31	99.3%	28,024	817	0.9956
	**Pilot 1 mean**	**76,541,081**	**3,300,867**	**4.4%**	**34.4%**	**75.4%**	**35.75**	**97.7%**	**26,611**	**788**^**e**^	**0.9930**
Pilot 2	SA1000	77,069,730	8,387,491	10.9%	9.5%	57.3%	44	98.4%	27,921	2,483	0.9915
	SA1001	85,479,934	3,551,500	4.2%	11.4%	74.2%	67	98.7%	27,694	2,318	0.9939
	SA1002	92,542,846	4,674,919	5.1%	15.5%	70.1%	65	98.8%	27,886	3,286	0.9941
	SA1006	83,545,692	4,002,665	4.8%	18.1%	74.5%	59	98.4%	27,446	2,442	0.9927
	SA1010	87,939,484	4,445,502	5.1%	14.5%	71.0%	62	98.6%	27,295	1,782	0.9935
	SA1011	82,377,158	7,810,714	9.5%	11.6%	49.2%	40	98.5%	27,484	2,717	0.9887
	SA1025	81,405,650	2,498,412	3.1%	10.0%	87.8%	63	99.3%	28,696	2,676	0.9934
	**Pilot 2 mean**	**84,337,213**	**5,053,029**	**6.1%**	**12.9%**	**69.2%**	**57.14**	**98.7%**	**27,775**	**2,529**	**0.9925**

^a^Total number of DNA fragments including: mapped, unmapped and duplicate reads.

^b^Limited to non-duplicate reads on autosomes, as calculated by GATK Unified Genotype.

^c^Limited to XX autosomal SNPs identified at the 99% VQSR threshold.

^d^Concordance at heterozygous and homozygous non-reference positions as compared to Illumina OmniExpress or 550K.v2 SNP arrays.

^e^Fewer average singletons as a result of including closely related individuals in Pilot 1. See Additional file 1: Table S1 for individual data.

Two samples (SA006 and SA035) displayed a high percentage of duplicate reads (54% and 78%) (Additional file 1: Figure S2, Table 1). To understand whether SA006 and SA035 had high duplicate rates due to low human DNA input or whether there were other issues with read data, we examined the distribution of mapping quality for all uniquely mapped reads for each sample. These two samples had the lowest numbers of mapped reads and the lowest proportion of reads with mapping qualities ≥ 30 (35.2% and 68.6%, respectively, Additional file 1: Figure S3). The remaining Pilot 1 samples had higher effective coverage and ~80% of reads with mapping qualities ≥30. This difference is unlikely to be due to divergence from the reference because we observed no systematic differences in mapping quality metrics between the European- and Bantu- admixed Family 1 and the KhoeSan Family 2. Due to lower mapping rates, SA006 and SA035 displayed overall lower mapped coverage than the other samples. However, 90% of target sites were covered at a depth of at least 10x for all individuals except SA006 and SA035 (Additional file 1: Figure S2). The average percent of unmapped reads was higher for saliva-derived exomes compared to six HGDP San samples sequenced using DNA obtained from cell lines (Additional file 1: Table S1). However, the primary difference in sequencing efficiency between saliva- and cell-line derived DNA results from differences in the mean rate of duplicate reads: Pilot 1, 34.4%; Pilot 2, 12.9%; HGDP, 9.8%. Pilot 1 likely has a higher duplicate rate due to lower DNA quality (see below).

### Read quality

**Figure 2:**
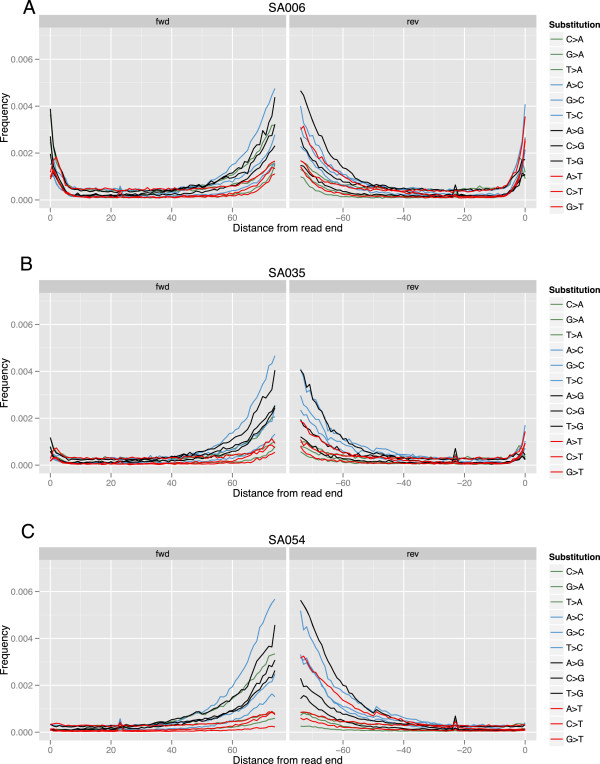
**Assessment of base substitutions from mapped reads.** Each mapped read was compared to the genome reference sequence to assess patterns consistent with DNA degradation. At each of the 75 positions along a read, we plot the frequency of substitution types, for both the forward (left) and reverse (right) reads from each read-pair. Analysis was limited to 1 million reads from chromosome 1; all raw reads are plotted. Three individuals with varying levels of substitution errors are shown: **(A)** SA006 with overall higher substitution rate and an excess of purines at the start of the first read, **(B)** SA035 with a slightly elevated substitution rate and excess of purines at the start of the first read, and **(C)** SA054 with a low substitution rate and no bias at the beginning of the first read. The additional five Pilot 1 individuals tended to resemble SA054 (Additional file 1: Figure S4). Removal of reads with any soft-clipping substantively reduced the mis-incorporation rate for SA006 and SA035.

We hypothesized that the difference in mapping quality among samples could be due to different levels of DNA damage. To test this hypothesis, we analyzed the distribution of mismatches along the reads by comparing each read to the human reference sequence after mapping. If the genomic DNA had been degraded before shearing, for example due to variable storage conditions, one would expect an increase in mismatches at the ends of the reads; specifically, an excess of thymines at the 5′ end of the read and an excess of cytosines at the 3′ end of the read, similar to what is seen in ancient DNA [19-21]. However, for SA006 and SA035 we observe an increased rate for all types of substitutions at the beginning of the reads, with the highest rates for those towards the purines G and A (Figure 2). Reads from SA006 in particular show a pronounced increase, for example a ~10 fold increase in the rate of A - > G substitutions in the first position compared to positions further along the read. This pattern is absent from all other samples in Pilot 1, with the exception of SA051, which also shows a slight increase at the first base (Additional file 1: Figure S4). We also observe an overall increase in substitution rates towards the end of the reads, which is shared across all samples and consistent with the increased rate in sequencing error with increasing number of sequencing cycles. The pattern of mismatch rates does not support a hypothesis of simple degradation.

### Genotype and variant statistics

Variants were called using the Genome Analysis Tool Kit (GATK) and selected using the Variant Quality Score Recalibration (VQSR) procedure with cutoffs set such that 99% of variants also found in the 1000 Genomes Omni2.5 and HapMap3 SNP training set were retained [22-24]. We identified 82,093 variants, with a transition/transversion ratio of 3.14. On average, within the target regions, each individual had a genotype call at 98% of sites variable in the 15 sample dataset (Table 1). Singleton counts varied from 657 to 3,286 autosomal sites, excluding the two daughters in Family 1 (Table 1). We computed genotype concordance for 12 individuals (sufficient DNA was not available for SA011, SA012, SA051) based on data from the Illumina OmniExpress or 550 K.v2 SNP arrays [2]. Non-reference (NR) concordance, that is concordance only at heterozygous or non-reference homozygous genotypes, was calculated using GATK [24,25] and concordance exceeded 98% for all individuals genotyped.

### Novelty compared to 1000 genomes project

**Figure 3:**
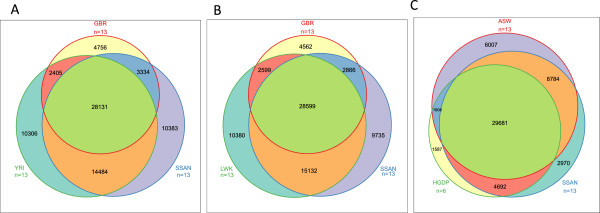
**Novelty compared to 1000 genomes project.** We compared the number of nonreference variants in the South African KhoeSan [SSAN] with **(A)** 1000 Genomes Yoruba samples [YRI], **(B)** eastern African Luhya [LWK], and **(C)** Namibian San from HGDP and African-Americans [ASW]. Sites that were included in this analysis required the presence of genotype information for at least 95% of the individuals in the joint dataset. The exome data from ASW and LWK was derived from 1000 Genomes Project, Phase 1 – March 17, 2012 release, from which 13 individuals were randomly sampled. The Venn diagram illustrates the number of shared and unique nonreference variants among populations. The Vennerable package in R was utilized for plotting purposes.

We compared 13 KhoeSan exomes from our study (excluding children SA011 and SA012), to exomes sequenced as part of the 1000 Genomes Project (1000G) [23], HGDP Namibian San, and San Nimblegen exomes from Schuster et al. [15]. We chose three populations of African ancestry for comparison: ASW, African-Americans from the Southwestern United States; LWK, Luhya from Kenya; YRI, Yoruba from Nigeria; and GBR, from Great Britain to represent European ancestry. Since the 1000G dataset contained many more individuals than our KhoeSan dataset, these populations were randomly down-sampled to 13 individuals for comparison. We note that these disparate datasets were processed using different pipelines, in some cases involving multiple-sample calling and imputation with a large number of other exomes, with varying degrees of coverage and sample relatedness. Between 28,000-29,000 variants appear to be common to all 5 populations (i.e. between 38%-53% are shared in each three-way comparison) (Figure 3). The South African ≠Khomani San appear comparable to the Yoruba and Luhya populations in terms of the number of private SNPs yet share slightly more variants with GBR than either other sub-Saharan African population. This reflects the degree of recent European admixture in the ≠Khomani. In order to compare two KhoeSan populations, we used 6 Namibian HGDP San exomes sequenced on the same Agilent Platform to >70x coverage [Martin et al, in preparation] and included African-Americans in an attempt to control for recent gene flow from Bantu-speaking and European groups (Figure 3C). The Namibian and South African populations share only 4,692 unique variants that are not also found in the ASW. This may reflect the small sample size, or that the KhoeSan populations in the north vs. south Kalahari remain highly differentiated [26,27]. Given the high concordance between our exome sequence data and the Illumina SNP array, we believe that the high genetic diversity of the South African exomes is not an artifact caused by high false positive rates.

### Population differentiation of the KhoeSan

**Figure 4:**
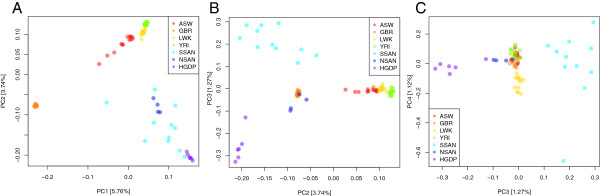
**Principal components analysis of 61,000 exonic SNPs in the ≠Khomani San and other African populations.** Exomes from 1000 Genomes Phase 1, Schuster et al. [15], and HGDP San were combined with the ≠Khomani San (related samples from Families 1 and 2 were removed). 5.76% of the variance is explained by PC1, 3.74% by PC2, 1.27% by PC3, and 1.12% by PC4. PC1 and PC2 separate Africans from Europeans, and western Africans from southern Africans, respectively **(A)**. The three KhoeSan populations drive PC3 and PC4 **(B and C)**, supporting prior descriptions of strong differentiation among Kalahari KhoeSan groups [27], and indicating even sub-structure within the ≠Khomani San samples.

We performed principal component analysis (PCA) on the unrelated ≠Khomani KhoeSan (Additional file 1: Figure S1), Schuster et al. [15] Namibian San, and HGDP Namibian San exomes, along with several different populations from the 1000 Genomes Project using smartpca from the EIGENSOFT software package [28] (Figure 4, Additional file 1: Figure S6). PCA clearly differentiates the populations included in this study. PC1 separates the African populations from the European population (GBR). PC2 separates the populations of western African ancestry (LWK and YRI) from the southern African populations (SSAN, NSAN, and HGDP San). PC3 separates the northern from the southern Kalahari KhoeSan populations, suggesting there is substantial substructure among these groups. PC4 separates a single SSAN individual from both the HGDP San and the rest of the ≠Khomani KhoeSan individuals. This individual belongs to Family 2, so the PCA was revised to include the remaining two Family 2 individuals (1^st^ cousin/half-sibling relationships, Additional file 1: Figure S1) in order to assess whether SA051 was an outlier, however PC4 still strongly separated all Family 2 individuals from other ≠Khomani (Additional file 1: Figure S6).

### HLA and KIR

The *HLA* and *KIR* loci include some of the most polymorphic genes in the human genome and are functionally involved in the immune system and reproduction [29,30]. Contributing to *HLA* and *KIR* polymorphism are inter-locus recombination and gene duplication, factors rendering these loci difficult to analyze with genomic-scale data, but among the most stringent for assessing its validity. We analyzed the three highly polymorphic *HLA class I* genes, *HLA-A*, -*B* and -*C* (6p21), and the *KIR* locus (19q13.4), which has variable content of four to thirteen polymorphic genes. Despite using a highly conservative strategy to remove read-pairs that did not map exclusively to one of the targeted loci, genotypes were obtained for 4,070 *HLA class I* and *KIR* SNPs for the fifteen individuals studied (Tables 2 and 3, Additional file 1: Table S2, Additional file 1: Table S3). Sufficient read-depth (at least 20 for homozygous positions and 10 for heterozygous positions) was obtained for determination of all the *HLA class I* and *KIR* alleles present, with exception of *HLA-A* and *-B* from individual SA006. Fourteen of the individuals were genotyped using standard methods for *HLA class I* and eight for all of the *KIR*. When comparison of the methods was restricted to the individuals with high genome-wide coverage, all but ten SNPs were concordant with standard genotyping from these samples (Tables 2 and 3), validating the sensitivity and specificity of our analytical approach (Methods). Moreover, all 39 discordant SNP genotypes from the two low-coverage individuals (SA006 and SA0035) occurred in clusters of low read depth where only one or neither allele was represented. Thus, following stringent filtering there were no false positive genotypes for any of the *HLA class I* or *KIR* SNPs. In total, there were 36 distinct *HLA class I* and 91 *KIR* alleles present, including thirty-one previously unknown *KIR* alleles that were discovered by analysis of the exome-sequencing data and independently verified by standard cloning, sequencing and family study.

**Table 2:** HLA and KIR validation

	**Exome sequencing**			**Standard genotyping**		**Standard genotyping (excluding SA006 & SA035)**	
	**SNPs vs HG19**^**a**^	**Alleles**^**b **^**Present**		**SNPs vs HG19**	**Concordance rate (%)**	**SNPs vs HG19**	**Concordance rate (%)**
		**Known**	**Novel**				
KIR (13 genes)	1469	91	31	955	99.99	670	99.99
HLA class 1 A	690	16		690	99.98	619	100.00
HLA class 1 B	925	12		925	99.99	745	99.99
HLA class 2 C	986	8		986	100.00	814	100.00

^a^Non-reference single nucleotide polymorphisms.

^b^Unique coding sequences.

**Table 3:** HLA and KIR validation for SA006 and SA035

	**Non-reference SNPS**			
	**SA006**^**a**^		**SA035**	
**HG19 ref**	**Present**	**Correct**	**Present**	**Correct**
A*03:01	37	12	19	19
B*07:02	71	58	34	34
C*07:02	40	39	50	50

^a^It was not possible to obtain the HLA-A and -B genotypes from exome data of SA006.

### Saliva metagenomes

Although exome capture proved an efficient method of sequencing primarily human DNA, each sample also contained more than a million unmapped reads (Table 1). We hypothesized that these unmapped reads might represent non-human DNA carried through the saliva extraction. Although we obtained useful results, with high concordance to SNP genotyping arrays, such microbial contamination may contribute to lower effective coverage levels. We therefore subjected these unmapped reads to an independent quality control procedure and used a fragment recruitment approach described by Rusch et al. [31] to identify homologs of non-human reference genomes among a combined pool of 24,139,131 high-quality unmapped reads (Figure 1). To estimate the number of species that are detected, we applied a recruitment threshold based on the 95% average nucleotide identity threshold that is commonly used to define microbial species [32].

**Figure 5:**
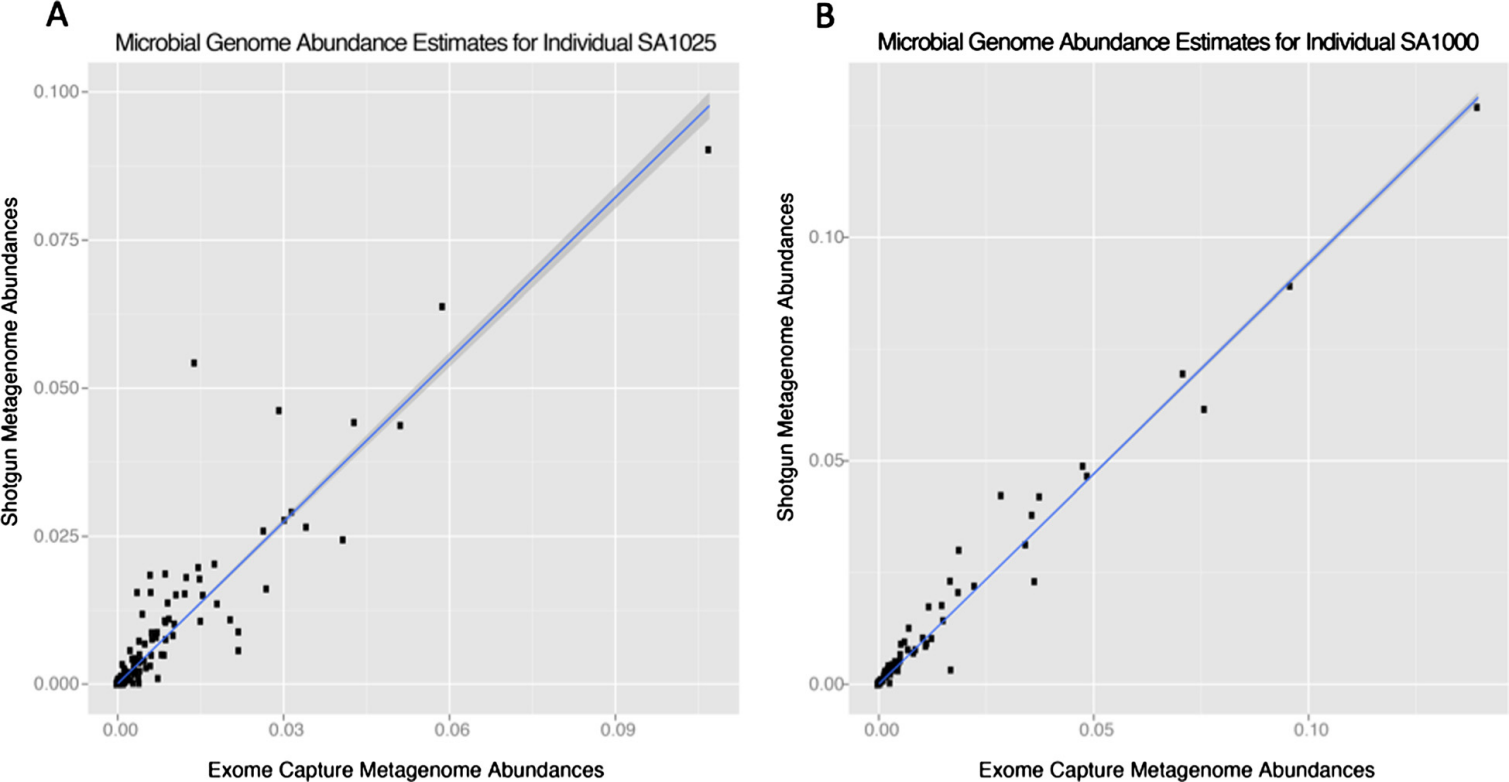
**Comparison of saliva microbiome frequencies from full genome and exome-capture sequencing.** Estimates of the relative abundance of saliva microbiota obtained via exome capture (x-axis) strongly correlate with those obtained from shotgun metagenomes produced from the same sample (y-axis). The above dot plots illustrate this result for two KhoeSan individuals involved in our study: **A)** SA1000 and **B)** SA1025. Each dot represents a genome. A linear model representing the relationship between exome-capture and non-capture estimates of relative abundance is shown in blue; the variance in the predictions from the model are shaded in grey. A Spearman correlation test indicates that this relationship is very strong (rho > 0.65; p < 2.2e-16).

Across all 15 sequenced exomes, we identified 1,835,400 high-quality reads (7.6%) that map to the genomes of 1,153 non-human species. The distribution of the number of recruited reads per genome indicates that a small number of genomes recruit a large number of reads with most genomes recruiting an insignificant fraction of the reads. For example, after normalizing the number of reads recruited per genome by reference genome size, the 100 most abundant genomes recruit 98.3% of the reads. Generally, the genomes that recruit the most reads are well-described oral commensal microbiota (Table 4), such as *Neisseria subflava*, *Rothia mucilaginosa*, *Neisseria flavescens*, *Veillonella dispar*, and *Prevotella veroralis*. The recruitment of reads across the length of these genomes suggests that their detection is not an artifact of a genomic subsequence that shares similarity with the human genome (e.g. Additional file 1: Figure S7.) We verified this by comparing the per genome relative abundance distribution estimated through analysis of these exome-captured metagenomes to the corresponding distribution estimated through analysis of non-capture metagenomes for two of the samples subjected to additional sequencing without exome capture (SA1000 and SA1025). Specifically, we find a significant, positive correlation (Spearman’s rho > 0.65; p-value < 2.2e^-16^) between the relative abundance estimates calculated with the two sequencing approaches for both samples (Figure 5), indicating that analysis of exome-captured metagenomes produces saliva community structure and abundance estimates that are surprisingly consistent with estimates from traditional shotgun metagenomic sequencing of saliva communities.

**Table 4:** KhoeSan saliva microbiome abundance by read threshold

	**No coverage**^**1**^	**75% Read coverage**^**2**^	
**Genome**	**50% Identity**	**80% Identity**	**95% Identity**
*Neisseria subflava*	0.062	0.077	0.13
*Rothia mucilaginosa*	0.051	0.062	0.076
*Neisseria flavescens*	0.031	0.039	0.063
*Streptococcus parasanguinis*	0.038	0.046	0.063
*Prevotella melaninogenica*	0.047	0.054	0.059
*Veillonella dispar*	0.041	0.049	0.056
*Prevotella veroralis*	0.027	0.031	0.029
*Streptococcus salivarius*	0.016	0.019	0.028
*Granulicatella elegans*	0.02	0.025	0.027
*Fusobacterium periodonticum*	0.015	0.012	0.024

^1^The genome-length-corrected relative abundance calculated using a 50% identity fragment recruitment threshold.

^2^The genome-length-corrected relative abundance calculated using fragment recruitment thresholds of 80% or 95% identity across at least 75% of the read.

Some of the abundant KhoeSan saliva microbiota are known contributors to oral disease. For example, *Prevotella melaninogencia* (recruits 5.9% of unmapped reads after correcting for genome length) is associated with rapidly progressing periodontitis lesions [33]. Similarly, *Streptococcus parasanguinis* (6.3%) is a primary colonizer of human teeth and contributes to dental plaque formation [34]. *Granulicatella elegans* (2.7%), an oral commensal associated with infective endocarditis [35], is also found in high abundance among the KhoeSan. We also specifically ascertained the presence of several biomedically important organisms, some of which may exist at relatively low abundance. For example, the *Porphyromonas gingivalis* genome, which represents organisms implicated in periodontal disease and has been linked to rheumatoid arthritis [36] and heart disease [37], recruits a relatively large fraction of reads from all individuals (1.68%). Conversely the *Campylobacter rectus* genome, which is also associated with periodontitis [38], recruits a relatively small fraction of reads (0.24%). Only 8 reads (2.3 × 10^-4^% of genome length-corrected recruitments) were recruited with high fidelity to the genome of *Mycobacterium tuberculosis,* the causative agent of tuberculosis, a disease that is common in the Northern Cape region of South Africa [39]. These reads map with equally high fidelity to the genomes of other Actinobacteria, suggesting that they may be homologs of ancient and highly conserved Actinobacteria sequences and are not necessarily representatives of the *M. tuberculosis* genome. Robust detection of *M. tuberculosis* from saliva-derived exome capture sequence data requires additional experimentation and validation.

**Figure 6:**
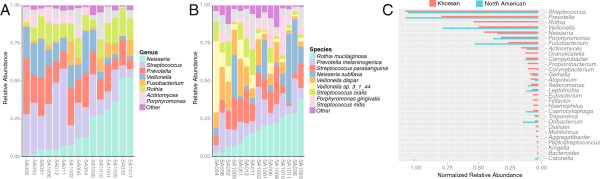
**Differences in taxon ranks between South African samples and human microbiome project. A, B)** Oral microbiome structure varies among the KhoeSan. Each of the above stacked bar plots illustrates the relative abundance (y-axis) of the most abundant oral microbiota at the **A)** genus, and **B)** species levels for each of the 15 KhoeSan individuals (x-axis). Relative abundance was measured as the fraction of high-quality reads that were recruited to a microbial genome of a particular taxonomic rank using conservative recruitment settings (Methods). Only the nine most abundant groups for each taxonomic level are illustrated for visualization purposes, with the remaining taxa being grouped into the ‘Other’ category. **C)** KhoeSan (red) and healthy North American (blue) saliva microbiomes differ in their community structure. In this bar plot, the normalized relative abundance, which is a taxon’s median relative abundance detected within a population divided by the maximum relative abundance detected within a population, is shown for bacterial genera that are detected in either of the two populations. Genera are ordered by their median relative abundance across the KhoeSan. Notable differences between the populations are those where the taxon is abundant in the KhoeSan and effectively undetected in the North Americans, especially *Rothia*.

The predominant saliva microbiota differ in their relative abundance across the KhoeSan (Figure 6). To assess whether population structure based on saliva microbiome diversity exists among the KhoeSan, we clustered individuals based on their phylum-, genus-, or species-level relative abundances. We find only moderate support for the existence of discrete clusters among the KhoeSan, with a maximum average silhouette width of 0.46 (genus-level clustering). Following [40], this suggests that saliva microbiome diversity varies among the KhoeSan along a gradient. We subjected the microbiome abundances of the KhoeSan samples to Principal Components Analysis (PCA) to identify those taxonomic groups that most strongly differentiate the samples along this gradient (i.e., maximum PCA loadings). At the phylum-level, KhoeSan saliva samples are principally separated by their relative composition of Proteobacteria, Firmicutes and Bacteroidetes (Additional file 1: Figure S9). The relative abundance of *Neisseria*, *Streptococcus*, *Prevotella*, and *Veillonella* primarily differentiate samples at the genus-level (Figure 6A), while *Rothia mucilaginosa*, *Neisseria subflava*, *Veillonella* spp., and *Streptococcus mitis* are some of the most variable species amongst the KhoeSan (Figure 6B).

### North American versus south African oral microbiomes

We then compared the diversity of the KhoeSan oral microbiome to the diversity observed in a recent and extensive survey of healthy North Americans in the Human Microbiome Project (N = 294) [41]. This prior HMP work was conducted through analysis of small subunit ribosomal RNA (i.e., 16S rRNA) gene sequences that were taxonomically annotated to the genus level. We used these sequences to calculate genus-level, genome length-normalized relative abundances for each North American microbiome. We used the taxonomy associated with each genome in our fragment recruitment database to calculate genus-level, length-normalized relative abundances for each KhoeSan microbiome. Comparing each population’s median relative abundance for each genus, we find that most taxa exist at similar abundance levels in the two populations (Spearman’s rho = 0.91, p-value < 2.2e-16). However, there are five genera that are present in relatively high abundance (Bonferroni-corrected Wilcoxon rank sum test p < 0.01) in the KhoeSan and effectively undetected among the North Americans given the level of discovery in the HMP (Figure 6C): *Rothia*, *Granulicatella*, *Haemophilus*, *Eubacterium*, and *Filifactor*. Most notable among these is *Rothia*, which is the third most abundant genus in the KhoeSan and contains *Rothia mucilaginosa*, a known oral opportunistic pathogen that has been linked to systemic diseases [42,43].

## Discussion

### Population history

The extremely high genetic diversity in the KhoeSan, estimated from genome-wide SNP arrays and the “Bushman” genome, has renewed interest in understanding the population history of southern Africans [2,15,26,27]. Comparatively few genomic sequences are publicly available (6 individuals total) from the KhoeSan, and ascertainment bias on many of the standard SNP arrays may strongly skew estimates of genetic diversity in these populations. We have generated 15 exomes and 2 genomes from the South African ≠Khomani San greatly expanding the number of genomic sequences available. Estimates of genetic diversity from these South African individuals are comparable to genetic diversity from the Yoruba from Nigeria or Luhya from Kenya (Figure 3). While we do not find a higher number of private SNPs in the KhoeSan, this may be biased due to endogamy among the ≠Khomani San and differences in coverage or SNP calling/imputation pipelines between 1000 Genomes and our procedure (Figure 1). Heterozygosity and singleton identification remain highly sensitive to coverage and calling pipelines thus making direct cross-study comparisons difficult. However, for common SNPs, we show that the KhoeSan strongly differentiate from all other human populations in structure analyses; the KhoeSan and Europeans fall at opposite ends of the 1^st^ principal component, while western and eastern Africans fall at intermediate points on this axis. Furthermore, we find substantial sub-structure among the South African and Namibian KhoeSan, despite recent gene flow from Bantu-speaking groups and Europeans into the ≠Khomani, !Kung and Tuu populations.

### Sample quality

Two of our samples had demonstrably lower mapping quality and coverage, SA006 and SA035. We consider three possibilities for these characteristics. First, it is difficult to identify the proportion of human DNA versus microbial or other non-human DNA in a saliva aliquot. If these two samples had by chance a lower volume of human DNA input for the exome capture reaction, then there would be fewer opportunities for human DNA to bind to the specific probes and the library would likely result in a higher number of duplicate read pairs. SA006 and SA035 do display an increased duplicate rate (54%, 78% respectively), but SA008 also displays high duplicate rate with minimal effect on mapping quality. Additionally, poorer mapping quality might be expected if the microbial reads map to the human genome, perhaps due to near sequence identity between some portion of the human and microbial genomes [44].

A second possibility is that the total amount and quality of the human DNA input initially may have been sufficient, but the presence of non-human substances such as residual tobacco or bacterial DNA may have acted as inhibitors, preventing normal binding to human probes. Third, the DNA in these two samples may have been more degraded than the other six Pilot 1 samples. However, although we do observe an increase in substitutions at the start of the reads for SA006 and SA0035, we find no evidence of an ancient DNA degradation pattern in the post-capture sequence data. While the listed possibilities appear unlikely, it is possible other patterns of degradation occur, in relatively young DNA extractions, which have not been reported in the literature.

### Oral microbiome from exome sequencing

Approximately 5.1% of the sequence data generated did not map to the human genome. Using a phylogenetically diverse set of reference genomes and a fragment recruitment approach, we identified those unmapped reads that are homologs of regions in non-human genomes. We find that most of the reads map to genomes of well-described commensal microorganisms of the human mouth, suggesting that this sequencing platform produces relevant information about the human oral microbiome. We also find that analysis of exome-capture metagenomes produces microbiome diversity estimates consistent with those obtained from non-exome-capture metagenomes, indicating that this platform can be used to reliably quantify microbiome diversity and abundance. We note that other capture technologies or probe designs may result in fewer off-target reads, and a corresponding reduction in the ability to analyze the microbiome [45,46]. Additionally, different saliva collection kits or the use of pre-collection mouth washes may effect the yield of microbial-derived sequences.

The large fraction of non-human sequences that do not map to our reference genomes are likely low quality and degraded sequences or are reads from organisms that are outside of the bounds of the phylogenetic diversity sampled in our reference database, such as viral genomes. The size of this fraction may be exacerbated by the relatively conservative alignment thresholds applied during our analysis. Our ability to detect oral commensals indicates that this human exome sequencing platform provides the added benefit of being able to assay biogeographic patters of oral microbiome diversity. Given that many of the non-human reads can be mapped with high stringency to genomes of known pathogens, we hypothesize that this sequencing platform may be useful as a diagnostic tool for the detection of disease and that the data obtained may be used for inferring cryptic phenotypes of the sampled individuals (e.g., periodontitis status). Future studies that focus on the sensitivity and specificity of pathogen detection will be required to test this hypothesis.

As a cautionary note, one genome that recruits a substantial number of reads (9.4% of total reads) is *Beggiatoa sp. PS. Beggiatoa* have been found in sulphur springs, sewage contaminated water, and hydrothermal vents [47]; to date, no one has described the presence of *Beggiatoa* in the human mouth. We found that the *Beggiatoa*-recruited reads map to short, unassembled contigs that exhibit significant similarity to clone libraries of the human genome. Thus, we suspect that our detection of *Beggiatoa* is the result of low quality human reads that fail to align to the human genome reference sequence but do align to regions of the *Beggiatoa* genome. This observation highlights the importance of considering the effect of human genome contamination when using fragment recruitment to study the human microbiome.

### KhoeSan microbiome diversity

Understanding KhoeSan microbiome diversity and structure provides insight into the co-evolution of the human microbiome, given the ancient divergence of KhoeSan from other African populations. It additionally clarifies the effect of lifestyle on microbiome composition as most studies focus on individuals living contemporary Western lifestyles. Similar to studies conducted in Western populations [48,49], we find that the KhoeSan salivary microbiome is dominated by a small number of taxa, with the Firmicutes or Proteobacteria predominating, and exhibits high diversity within and between individuals. These observations suggest that the general structure of the KhoeSan salivary microbiome is generally similar to that found in Western individuals.

However, when evaluating differences in the relative abundance of genera associated with the KhoeSan and a population of healthy Americans, we identified several abundant taxa in the KhoeSan that were at very low abundance or undetected among the Americans. These differences in microbiome structure may be due to differences in (1) the evolutionary history of the populations, (2) demographics, or (3) host environment or lifestyle, including diet and access to health care. Given that we find many known pathogens among the most abundant members of the KhoeSan microbiome and that many of the differentially detected genera contain known oral pathogens (e.g., *Rothia*, *Granulicatella*, *Filifactor*), we speculate that the relatively limited access to dental care, antibiotics and/or absence of water fluoridation among the KhoeSan is driving most of the observed differences between populations. However, the biology of several of the differentially abundant genera is not well understood, especially in the context of the commensal oral microbiome (e.g., *Mobiluncus*), or is principally limited to the pathogenic members of the genus; such genera may contain species that played an important role in the coevolution between the KhoeSan and their salivary microbiome. This may include pathogenic organisms, such as *Aggregatibacter actinomycetemcomitans*, the causative agent of adolescent periodontal disease, which is common in those of African descent [50] and a member of a relatively abundant genus in the KhoeSan. Further study of the microbiomes associated with the KhoeSan and other diverse human populations (e.g., [51]), the microbiomic differences between these populations (e.g., [52,53]), especially across a variety of host physiological conditions, and the biology of commensal microbiota that are underrepresented in Western populations is needed to comprehensively differentiate the sources of variations observed between populations and to understand the coevolution between humans and their microbiome.

## Conclusions

We have demonstrated the ability to obtain high quality exome sequence data from saliva-derived human DNA. We show that even samples with low human DNA presence can be successfully captured using exome in-solution target probes. Additionally, after examining some of the most diverse human loci, we find that exon-capture is able to enrich and facilitate high-resolution analysis of highly polymorphic *HLA* and *KIR* genes from DNA extracted from human saliva. We also demonstrated that exon-captured DNA sequencing of saliva reveals insight into the structure and diversity of the oral microbiome.

## Methods

### Samples

Sampling of the ≠Khomani KhoeSan in Upington, South Africa and neighboring villages occurred in 2006. Institution Review Board (IRB) approval was obtained from Stanford University. Individuals who were still living in 2011 were re-consented under a modified protocol (IRB approved from Stanford University and Stellenbosch University, South Africa). ≠Khomani N|u-speaking individuals, local community leaders, traditional leaders, non-profit organizations and a legal counselor were all consulted regarding the aims of this research, prior to collection of DNA. All individuals consented orally to participation, with a second, local native speaker witnessing and were re-consented with written consent. DNA via saliva (Oragene® kits) and ethnographic information regarding self-identified ancestry (N|u, Nama, or ‘Coloured’), language and parental place of birth were collected for all participants.

### Exome capture

Library preparation and exome enrichment were performed as described in the Agilent SureSelect^XT^ Target Enrichment System for Illumina Paired-End Sequencing Library (Version 1.1.1, January 2011). First, purified DNA from saliva samples was concentrated to a volume compatible with the library preparation protocol. 3 μg of concentrated genomic DNA was fragmented to a median size of 200 bp using the Covaris-S2 instrument with the following settings: duty cycle 10%, intensity 5, cycles per burst 200, and mode frequency sweeping for 180 s at 4°C. The fragmentation efficiency was evaluated on the Agilent Bioanalyzer using DNA1000 chips. After end-repair and A-tailing, sequencing adapters were ligated onto the DNA fragments, followed by size-selection using SPRI beads (Agencourt AmPure XP) and PCR amplification. The amplification product was purified with SPRI beads and the quantity and quality was assessed using the Bioanalyzer DNA1000 chip. Five hundred nanograms of the adapter-ligated DNA library were concentrated to 3.4 ml, mixed with hybridization buffer and DNA blocker mix, and added to the SureSelect 50 Mb All-Exon capture probe library. The mixture was incubated for 24 hours at 65°C in a thermal cycler. The hybridization mixture was added to streptavidin-coated M-280 Dynabeads (Invitrogen) and incubated for 30 min at room temperature, with mixing. The beads were washed with 500 ml SureSelect wash buffer #1 for 15 min. at room temperature, and three times with 500 ml SureSelect wash buffer #2 for 10 min at 65°C. DNA was eluted with 50 ml SureSelect elution buffer for 10 min at room temperature and neutralized with 50 ml of SureSelect neutralization buffer. The captured product was purified with SPRI beads and amplified by PCR. The quality and concentration of the sequencing libraries was verified by the Bioanalyzer High Sensitivity DNA kit (Agilent). Indexed samples were pooled in an equimolar ratio and sequenced on the Illumina HiSeq2000 according to standard protocols. A similar procedure was followed for the Pilot 2 samples with the SureSelect 44 Mb All-Exon capture probe library.

### Read mapping and SNP calling

Illumina sequencing reads were mapped to the human genome reference sequence (GRCh37) following a standard pipeline informed by the best-practices as described by the 1000 Genomes project [24,54] (Figure 1). Pilot 1 reads were trimmed to be 75 bp in length; Pilot 2 reads were 101 bp in length. Reads were mapped and paired using bwa version 0.6.2 [55]. Unmapped reads were identified at this stage and processed via the metagenomic pipeline. Duplicate read pairs were identified using Picard (http://picard.sourceforge.net/). Base qualities were empirically recalibrated and indel realignment was performed jointly across all samples using the Genome Analysis Tool Kit (GATK) v1.6 [25]. BAM files containing only uniquely mapped reads with duplicates removed were analyzed by the program SAMStat [56]. Fraction of reads on target was determined using snpEff.

Sequencing reads from the samples described in Schuster et al. [15] were obtained from the short read archive and remapped to the GRCh37 assembly. The exome capture data from Schuster et al. was single end sequences obtained from the 454 pyrosequencing technology. Reads were mapped using the bwasw option in bwa version 0.5.9. Processing was performed as described above, with the exception of omitting the ‘homopolymer’ recalibration covariate and skipping the indel realignment step which is not supported for 454 reads.

### Read substitution bias

For Pilot 1, rates of nucleotide substitutions at each position along the reads were determined by comparing the mapped reads to their aligned human genome reference sequence. We analyzed the first 1 million reads mapped to chr1 for each sample, using only reads without any alignment indels or clipping (with a CIGAR string of ‘75 M’ in the BAM file). For each read, we retrieved the corresponding aligned reference sequence using its mapped chromosomal position in the BAM file. The rates for each nucleotide substitution type were then calculated as the ratio of the total number of observed changes of that type and the total number of reads, for each position along the reads. Because reads mapping to the reverse strand of the reference are reverse complemented in the BAM files, we performed the analysis separately for forward and reverse strand mapping reads. Reverse mapping reads therefore show the complementary substitution patterns at the 3′ end to the forward mapping reads at the 5′ end.

### Population differentiation

To perform principal component analysis we used SNP genotypes for individuals from several populations and the EIGENSOFT software [28]. We used 11 KhoeSan individuals from our dataset (excluding SA011 and SA012 from Family 1 and SA052 and SA054 from Family 2), 4 Namibian KhoeSan individuals from Schuster et al., 6 Namibian San (Ju|’hoansi) from the Human Genome Diversity Project (Martin et al, *in prep*. SRP036155) [57], and 13 individuals from each of the ASW, GBR, LWK, and YRI populations from the 1000 Genomes Project [23]. Closely related individuals were excluded from all datasets. Sample ‘ABT’ was excluded from Schuster et al.’s dataset since it clustered with the Bantu-speaking populations in their analyses. Individuals selected from the 1000 Genomes Project all had more than 20x coverage for at least 70% of exome targets. To account for differences in coverage and target regions, variants included in this analysis had genotype information for at least 95% of the individuals for a given analysis. VCFtools [58] was used to count the number of shared and private SNPs between populations.

### HLA/KIR calling

To analyze the whole-exome data, all read-pairs that mapped within hg19 coordinates, chr6:28702021-33392022, chr19:55228188-55383188 and chr19_gl000209_random, were extracted using SAMtools 0.1.18 [59] and split into separate fastq files for each individual. Read-pairs having more than five bases of quality score ≤3 were removed (FASTX Toolkit 0.0.13 [http://hannonlab.cshl.edu/fastx_toolkit/]). The analysis pipeline was designed to detect all known and any novel *HLA class I* and *KIR* SNP variants. Using Bowtie (version 0.12.7) [60] read-pairs were harvested by mapping with low-stringency to a given *HLA* or *KIR* gene (positive filter). To ensure specificity, pairs that mapped to any homologous gene or pseudogene were removed (negative filter). The remaining reads were then aligned to a final reference sequence and the SNP variants ascertained using SAMtools/bcf. Data used to generate filters and reference sequences was obtained from the ImmunoPolymorphism Database and a set of fully-sequenced *KIR* haplotypes [61-63]. To accommodate the high divergence of *HLA* exons 2 and 3, the final alignments were made to reference sequences matching individual *HLA-A*, *-B* and *-C* genotypes. *HLA-A*, *-B* and *-C* reference alleles were determined using bead-based sequence specific oligonucleotide probe hybridization and were described in [2]. The “-phase” function of SAMtools was used to attribute phase for local alignments where possible due to the close proximity of exons and/or presence of highly heterozygous sequence (e.g. exons 2 and 3 of *HLA class I*). Post-filtered read depth was used to determine presence or absence of the variable-content *KIR* genes. The *KIR* genes present and their alleles were determined for comparison of eight of the individuals using pyrosequencing methods as previously described [64]. Individual SNP genotypes were confirmed visually from independent alignments of the filtered reads, which were created using MIRA 3 [65,66]. All newly-discovered variants were confirmed for sequence and phase using standard Sanger sequencing plus one or more of pyrosequencing, DNA cloning or segregation in families.

### Metagenomic pipeline

We searched for genetic signatures of non-human organisms by adopting the fragment recruitment approach outlined by Rusch et al. [31] (Figure 1). We first trimmed reads and removed low-quality (i.e., reads that meet any of the following conditions: mean quality score less than 25, length less than 50 bp, presence of ambiguous bases) and exact duplicate reads from the set of those that did not map to the human genome using prinseq [67]. We then compared the remaining high-quality reads that did not map to the human genome to 1,285 genomes (Additional file 2: Table S4) obtained from the Joint Genomes Institute’s Integrated Microbial Genomes (IMG) database [44]. In the case of species that have multiple genome-sequenced individuals, we randomly selected a single individual genome to represent the species group. Each read was aligned to each genome using blast (blastall -p blastn -z 16300000000 -e 0.01 -m 8) and the resulting alignment summary statistics were used to infer each read’s taxonomy [45]. We explored several classification thresholds, including alignment e-value, alignment percent identity, and the ratio between the alignment length and the read length (i.e., coverage). We adopted several levels of threshold stringency to recruit reads to genomes for the purposes of inferring taxonomic diversity. Our thresholds were similar to those used in Rusch et al. [31], with modifications to account for the short length of our sequences.

In the lenient case (i.e., distant homology), a read was recruited to a genome if the two sequences shared a local alignment having at least 50% sequence identity. Using these parameters we identified 5,060,454 unmapped sequences (20.9% of total unmapped reads) that exhibit significant similarity to the collection of reference genomes. In the stringent case (i.e., recent homology), a read was recruited to a genome if the alignment covered at least 75% of the read and the sequences had at least 80% identity. Applying these thresholds found that 16.8% of the reads (N = 4,064,899) can be recruited by non-human genomes.

To conduct species-level binning, we applied the aforementioned coverage thresholds, but required that the read and target genome share at least 95% identity. In all cases of classification, we applied an e-value threshold of 10^-3^. We inferred a read’s taxonomy by transferring the taxonomic annotation of the genome sequence that produced the best alignment score while also passing the classification thresholds. If a read could not be placed into a species group based on the reference genomes, it was discarded from the subsequent diversity analyses. The IMG taxonomic annotations associated with the reference database genomes were used to assign species-level binned reads into genera and phyla.

To quantify genus-level saliva microbiome abundances among healthy Americans, we downloaded high-quality, taxonomically annotated V35 16S rRNA Roche amplicon sequences associated with 294 saliva samples from the Human Micorbiome Project (HMP) Data Analysis and Coordination Center (http://www.hmpdacc.org/). A prior study used the Ribosomal Database Project classifier (v2.2) with the default 032010 training set and taxonomy to annotate these sequences [49]. Genus-level taxonomic assignments were extracted for each sequence having a bootstrap statistic greater than 80%.

## Availability of supporting data

VCF files are available at http://ecoevo.stonybrook.edu/hennlab/data/. Raw read data can be downloaded from the short-read archive (SRP038015 for saliva derived exomes and genomes, and SRP036155 for HGDP San exomes). SNP variants have been deposited in dbSNP (SS 974432427-SS974514519) Novel KIR alleles have been deposited in Genbank and assigned Immuno Polymorphism Database nomenclature as follows:

JX523651 (3DL3*057), GQ924778 (3DL3*037), GQ924779 (3DL3*038), GQ924781 (3DL3*040), HM235773 (3DL3*041), JX523631 (2DL2*012), JX523638 (2DL5B*00803), JX523639 (2DL5B*018), JX523640 (2DS3*007), JX523642 (2DS5*012), HM358896 (2DS5*0502), JX523648 (2DP1*00103), JX523646 (2DP1*00202), JX523647 (2DP1*011), JX523644 (2DP1*012), JX523645 (2DP1*013), JX523643 (2DP1*014), JX523630 (2DL1*026 N), GU323355 (2DL1*022), JX523652 (3DP1*011), JX523655 (3DP1*012), JX523653 (3DP1*013), JX523654 (3DP1*014), JX523634 (2DL4*024), JX523637 (2DL4*027), GQ890695 (3DL1*070), GQ890697 (3DL1*071), GU323347 (3DL2*052), GU323348 (3DL2*053), GU323349 (3DL2*054), JX523649 (3DL2*063)

## Competing interests

MG is employed by Agilent Technologies. BMH and CRG hold stock in 23andMe, Inc. CDB is on the scientific advisory board of Ancestry.com. 23andMe, Inc. and Ancestry.com had no role in the study design, data collection and analysis, decision to publish, or preparation of the manuscript.

## Authors’ contributions

JMK, TJS, DB, PJN, ARM, MLC, MS and BMH analyzed data; CRG, EGH, and BMH collected and processed DNA samples; NN, AA, MG, XG, QF, YL, and XL generated genomic data; TJS and KSP performed metagenomic analysis; PP, MWF, KSP, CDB and JDW contributed to study design; JMK, TJS and BMH wrote the manuscript with input from all authors. All authors read and approved the final manuscript.

## Supplementary Material

Additional file 1: Table S1 Summary Statistics for HGDP San Exomes. **Table S2:** HLA alleles. **Table S3:** KIR alleles. **Figure S1:** Pedigree structure for sequenced individuals. **Figure S2.** Cumulative coverage across the Agilent target regions for Pilot 1 (A) and Pilot 2 (B) samples. **Figure S3:** Mapping quality for all reads. **Figure S4:** Assessment of base substitutions from mapped reads. **Figure S5:** Venn Diagram comparing ≠ Khomani San with Namibian exome samples. **Figure S6:** PCA with two relatives included. **Figure S7:** Distribution of mapped reads along the N. subflava genome. **Figure S8:** The phylogenetic distribution of three non-human exome capture sequences that map with high fidelity to Mycobacterium tuberculosis. **Figure S9:** The phylum-level structure of the oral microbiome structure varies among the KhoeSan. Click here for file

Additional file 2: Table S4 Species included in the microbial genome database. Click here for file
