# Supplementary material for: Exome capture from saliva produces high quality genomic and metagenomic data
Source: BMC Genomics. 2014 Apr 4;15:262. doi: 10.1186/1471-2164-15-262 (PMC4051168; doi:10.1186/1471-2164-15-262)
Supplement: Additional file 1: Table S1 — Summary Statistics for HGDP San Exomes. Table S2: HLA alleles. Table S3: KIR alleles. Figure S1: Pedigree structure for sequenced individuals. Figure S2. Cumulative coverage across the Agilent target regions for Pilot 1 (A) and Pilot 2 (B) samples. Figure S3: Mapping quality for all reads. Figure S4: Assessment of base substitutions from mapped reads. Figure S5: Venn Diagram comparing ≠ Khomani San with Namibian exome samples. Figure S6: PCA with two relatives included. Figure S7: Distribution of mapped reads along the N. subflava genome. Figure S8: The phylogenetic distribution of three non-human exome capture sequences that map with high fidelity to Mycobacterium tuberculosis. Figure S9: The phylum-level structure of the oral microbiome structure varies among the KhoeSan. [file 1471-2164-15-262-S1.PDF]

SUPPLEMENTARY MATERIAL

Exome Capture from Saliva Produces High Quality Genomic and Metagenomic Data

Jeffrey M. Kidd[1,2,*], Thomas J. Sharpton[3,4*], Dean Bobo[5], Paul J. Norman[6], Alicia R. Martin[1], Meredith L. Carpenter[1], Martin Sikora[1], Christopher R. Gignoux[7], Neda Nemat-Gorgani[6], Alexandra Adams[1], Moraima Guadalupe[8], Xiaosen Guo[9], Qiang Feng[9], Yingrui Li[9], Xiao Liu[9], Peter Parham[6], Eileen G. Hoal[10], Marcus W. Feldman[11], Katherine S. Pollard[3,12], Jeffrey D. Wall[12], Carlos D. Bustamante[1], Brenna M. Henn[1,5,§]

Supplementary Figures:

Figure S1 Pedigree structure for sequenced individuals
Figure S2 Cumulative coverage across the Agilent target regions
Figure S3 Mapping quality for all reads
Figure S4 Assessment of base substitutions from mapped reads
Figure S5 Venn Diagram comparing ≠Khomani San with Namibian exome samples
Figure S6 PCA with two relatives included
Figure S7 Distribution of mapped reads along the *N. subflava* genome
Figure S8 The phylogenetic distribution of three non-human exome capture sequences that map with high fidelity to *Mycobacterium tuberculosis*.
Figure S9 The phylum-level structure of the oral microbiome structure varies among the Khoesan.

Supplementary Tables:

Table S1 Summary Statistics for HGDP San Exomes
Table S2 HLA data
Table S3 KIR data
Table S4 List of Taxa Used in Metagenomic Analysis (*separate file*)

SUPPLEMENTARY MATERIAL

Table S1 Summary Statistics for HGDP San Exomes

Sample	Total Reads	Unmapped Reads	% Unmapped reads	% PCR Duplicates	Properly Paired	% Properly Paired
HGDP00987	84,355,854	1,452,143	1.72%	9.66%	81,943,050	97.14%
HGDP00991	88,790,056	2,275,297	2.56%	7.85%	84,862,816	95.58%
HGDP00992	79,270,474	1,400,201	1.77%	9.85%	76,845,818	96.94%
HGDP01029	86,406,698	1,880,970	2.18%	9.78%	83,240,246	96.34%
HGDP01032	86,687,656	1,817,084	2.10%	10.28%	83,626,708	96.47%
HGDP01036	92,438,170	1,794,121	1.94%	11.31%	89,373,204	96.68%

Table S2: *HLA alleles*

	HLA-A		HLA-B		HLA-C	
**SA006**	29:01	03:01	13:03	58:02	12:03	06:02
**SA008**	32:01	03:01	35:01	07:02	04:01	07:02
**SA011**	29:01	03:01	13:03	07:02	12:03	07:02
**SA012**	29:01	03:01	13:03	07:02	12:03	07:02
**SA035**	36:01	01:23	15:03	15:10	04:01	04:01
**SA051**	02:02	30:04	07:05	58:02	07:02	06:02
**SA052**	02:02	30:04	07:05	58:02	07:02	06:02
**SA054**	31:01	30:04	42:01	58:02	17:01	06:02
**SA1000**	33:01	68:02	41:01	53:01	04:01	17:01
**SA1001**	03:01	30:04	07:02	35:01	04:01	07:02
**SA1002**	11:01	30:01	35:01	42:01	04:01	17:01
**SA1006**	02:01	02:05	07:05	55:01	03:03	07:04
**SA1010**	03:01	68:01	58:01	58:02	06:02	x
**SA1025**	23:01	68:02	15:03	42:01	17:01	18:01

Shown are the alleles of HLA present in each of the individuals as determined from the exome sequences and verified by further sequencing (HLA-A, B, C). Alleles are named according to the ImmunoPolymorphism database (Robinson et al. 2010).

Table S3: *KIR alleles*

	3DL3		2DS2		2DL2		2DL5B		2DS3/5c	
**SA006**	*038	*01405	*004	*001	2*003	2*001	*002	*002	3*007	3*001
**SA008**	*040	*01403		*001	3*005	2*003				
**SA011**	*01403	*038	*001	*004	2*003	2*003		*002		5*012
**SA012**	*01403	*01405	*001		2*003	2*001		*002		3*001
**SA035**	*037	*038	*001	*004	2*00601	2*009	B*018	B*00803	5*0502	5*012
**SA051**	*057	*038	*001		2*003	3*001	B*018		5*0502	
**SA052**	*024	*038	*001	*001	2*003	2*012		B*00803		5*012
**SA054**	*041	*020	*001		2*003	3*005	B*018		5*0502	
**SA1000**	*00301	*008	*001		2*003	3*001				
**SA1001**	*01303	*01403	*001		2*001	3*002	B*00803		S3*00103	
**SA1002**	*01403	*01405	*001	*001	2*003	2*003	B*002	B*00803	S3*00103	S5*006
**SA1006**	*00102	*038	*001		2*003	3*002	B*00801			S5*002
**SA1010**	*01303	*038	*001		2*003	3*002	B*002		S3*00103	
**SA1011**	*005	***037**			3*001	3*018	B*00801			S5*002
**SA1025**	*00201	*038			3*001	3*001				

	2DL1		2DL4		3DL1/S1		2DS4		3DL2	
**SA006**	*00401	*00401	*00801	*006	*005	*007	*010	*004	*010	*008
**SA008**	*01202		*00103	*00802	*01502		*006		*00101	*054
**SA011**		*00401	*00802	*00801		*005		*010	*054	*010
**SA012**		*00401	*00802	*006		*007		*004	*054	*008
**Sa035**	*022		*013	*00103	*041	*070	*00101	*00101	*052	*00101
**SA051**	*022	*00302	*013	*00103	*041	*01502	*00101	*00101	*015	*063
**SA052**	*026N		*00102	*024	*002	*035	*00101	*00101	*00101	*015
**SA054**	*022	*00401	*027	*024	*041	*035	*00101	*00101	*052	*015
**SA1000**	*001	*004	*00102	*00103	*002	*070	*00101	*00101	*002	*023
**SA1001**	*001	*022	*00801	*013	*005	*041	*00101	*00101	*002	*015
**SA1002**	*010	*01201	*00801	*006	*007	*060	*00101		*008	
**SA1006**	*001	*01201	*00103	*00103	*01501	*008	*00101	*00101	*009	*053
**SA1010**	*002	*004	*00801	*01201	*001	*071	*00101	*00101	*001	*002
**SA1011**	2DS1*002		*00103		*020		*00101		*001	*006
**SA1025**	*001	*01201	*00801	*00801	*005	*060	*010		*010	

Shown are the KIR alleles present in each of the individuals as determined from the exome-capture sequences and verified by further sequencing. Alleles are named according to the ImmunoPolymorphism database (Robinson et al. 2010), and sequences discovered by the current study (shaded in purple) are denoted by local nomenclature. Clear boxes indicate that the gene is absent.

Supplemental Figures:

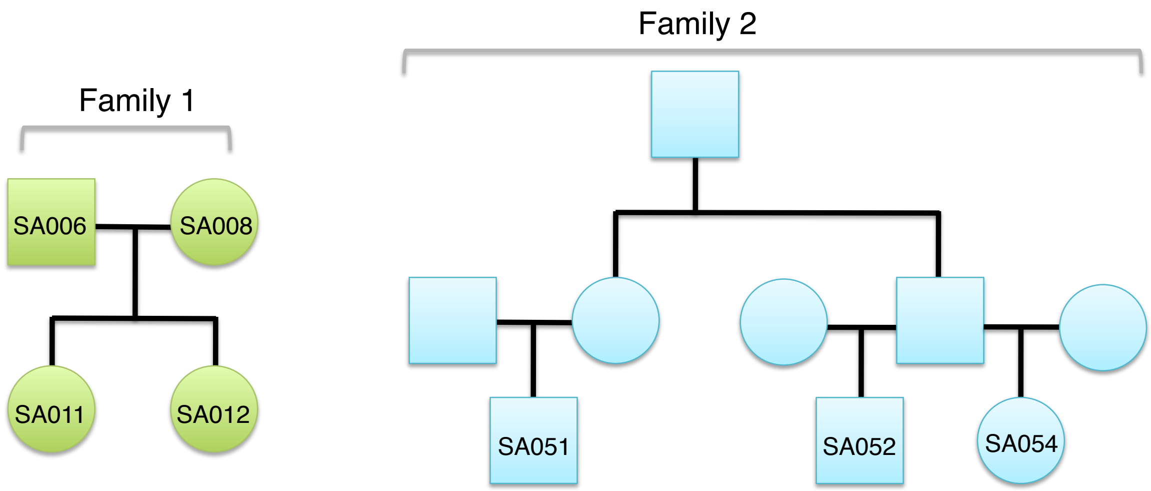

Figure S1 *Pedigree structure for sequenced individuals*. We selected 15 KhoeSan saliva samples from the Northern Cape Province, South Africa for exome capture using the Agilent 50Mb and 44Mb exome kits. The sample set consisted of four individuals from family 1, a quartet with two daughters, three individuals from family 2, an extended family of half-siblings and first cousins, and 8 purportedly unrelated individuals. Individuals ranged in age between 30-100 years.

A

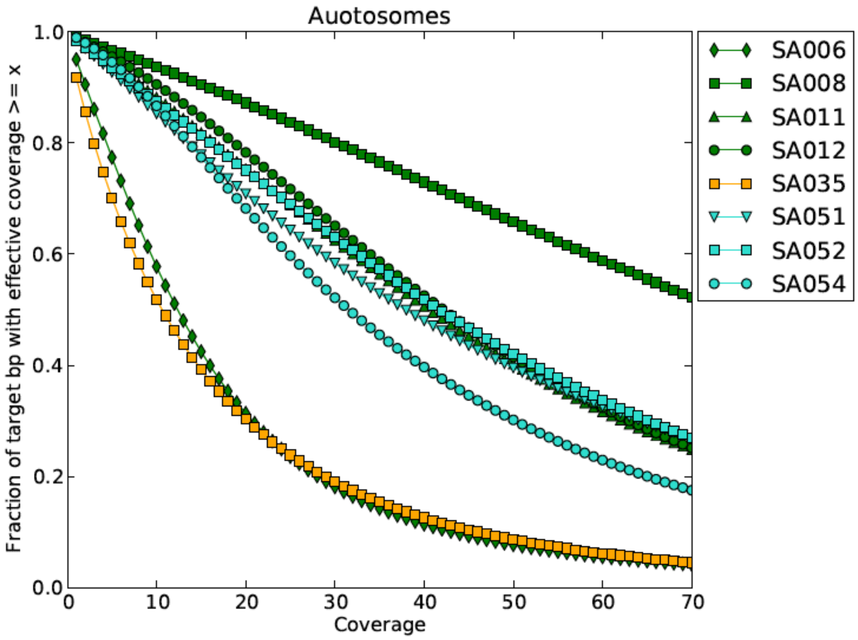

B

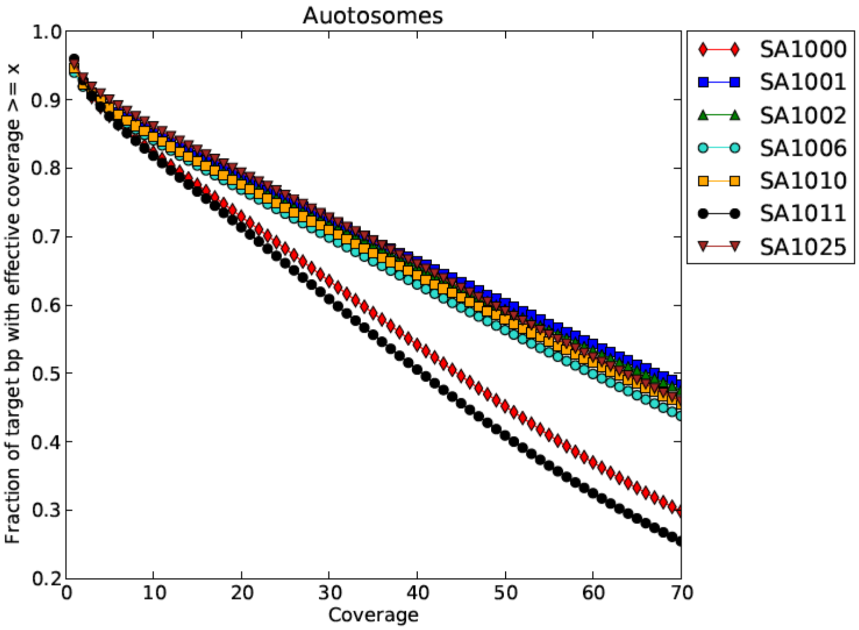

Figure S2 *Cumulative coverage across the Agilent target regions for Pilot 1 (A) and Pilot 2 (B) samples*. Effective coverage is based on non-duplicate reads used for SNP discovery by the GATK Unified Genotyper. Analysis was limited to the autosomes.

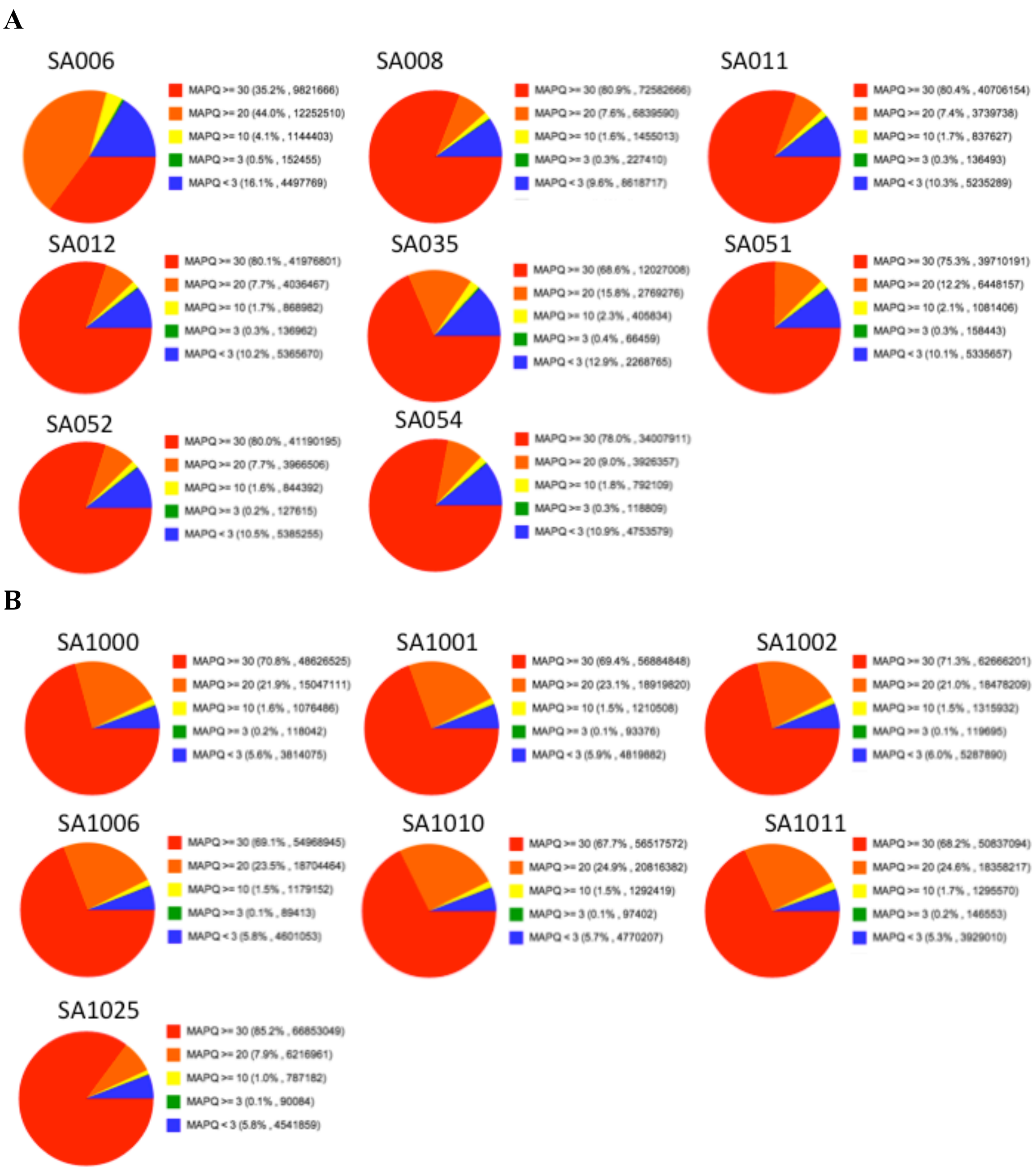

Figure S3 *Mapping quality for all reads*. Mapping quality proportions for all uniquely mapped reads from each individual sample from Pilot 1 (A) and Pilot 2 (B) are shown. Reads included both exome on-target capture and reads mapping off-target to the human reference genome. Mapping quality ≥30 is shown in red, ≥20 is orange, ≥10 is yellow, ≥3 is green, and ≤3 is blue.

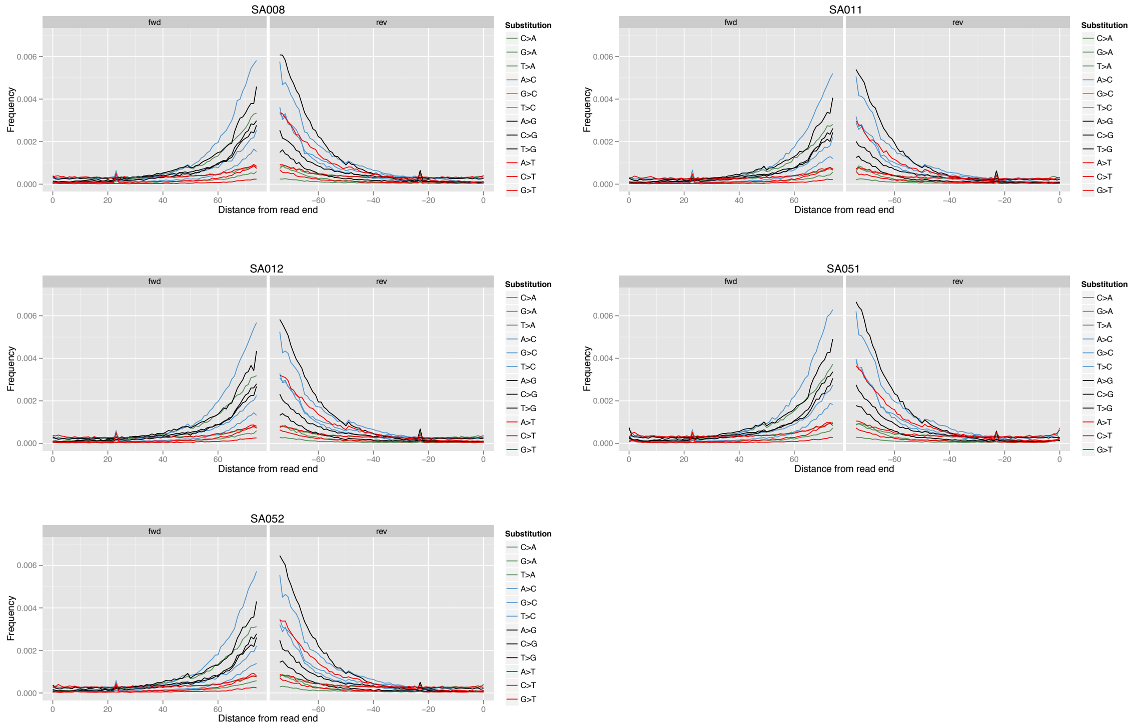

Figure S4 *Assessment of base substitutions from mapped reads*. Each mapped read was compared to the genome reference sequence to assess patterns consistent with DNA degradation. At each of the 75 positions along a read, we plot the frequency of substitution types, for both the forward (left) and reverse (right) reads from each read-pair. Analysis was limited to 1 million reads from chromosome 1; all raw reads are plotted.

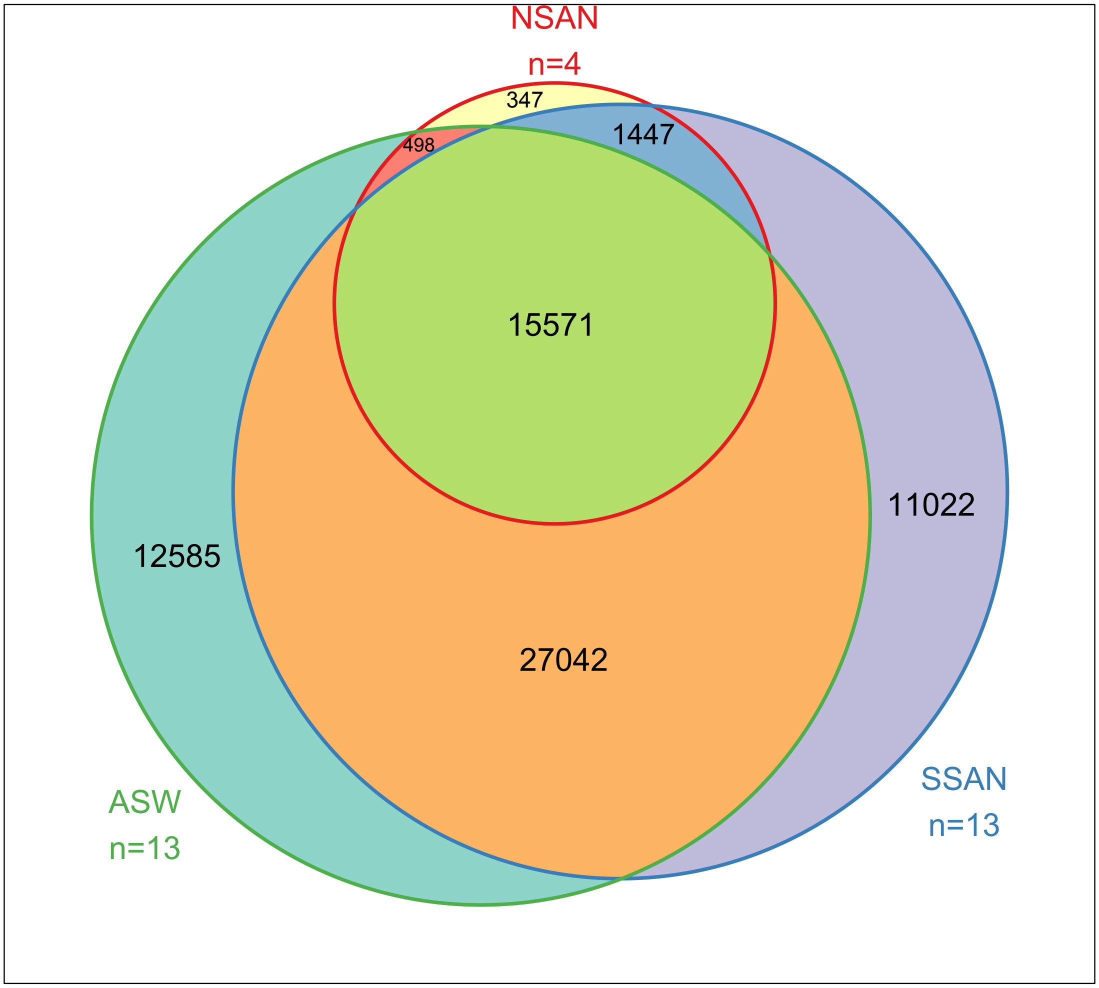

Figure S5 *Venn Diagram comparing ≠Khomani San with Namibian exome samples.* We compared the number of non-reference variants in our South African KhoeSan obtained with Agilent capture [SSAN] to the 4 Namibian San exomes from Schuster et al. obtained with NimbleGen capture [NSAN], along with African Americans [ASW] from the 1000 Genomes Project.

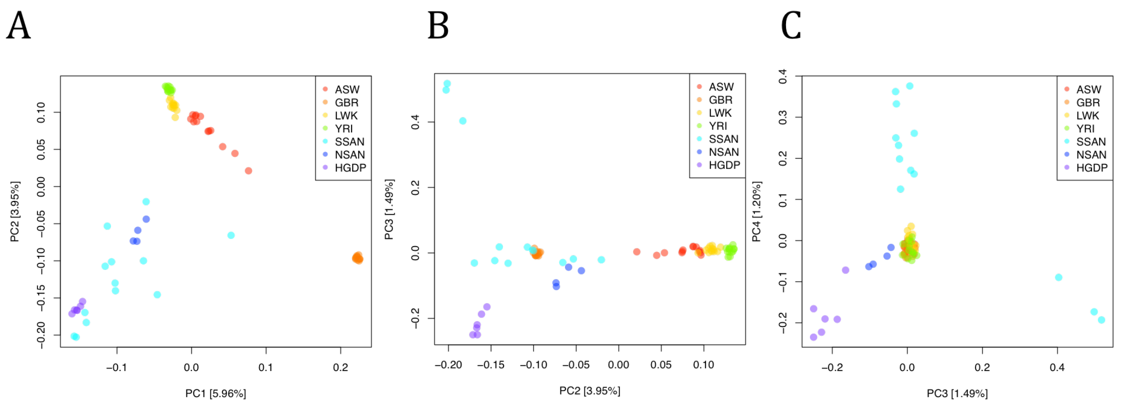

Figure S6: *PCA with two relatives included.* We repeated the Principal Component Analysis including two related samples from our ≠Khomani San population (SA052 and SA054 from Family 2) in order to assess whether SA051 was an outlier. All individuals from Family 2 separate from other ≠Khomani San along PC3 and PC4, suggesting differences in ancestry likely due to recent population structure in the southern Kalahari.

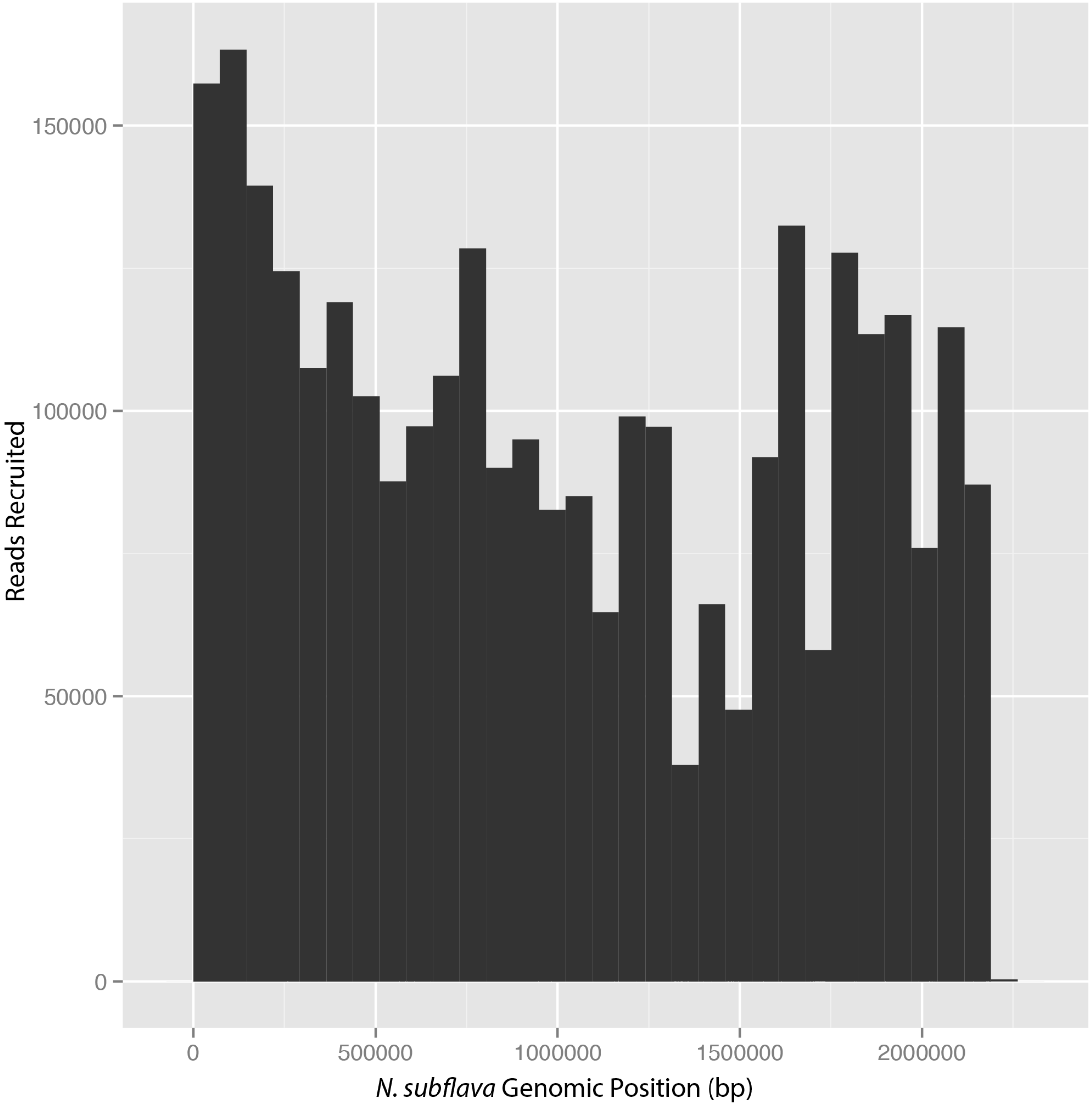

Figure S7 *Distribution of mapped reads along the N. subflava genome.* This histogram illustrates the number of reads that align to various positions of the *N. subflava* genome with 95% identity across at least 75% of the read's length. *N. subflava* genomic contigs were concatenated together and the number of reads that mapped to each base in the concatenated genome were counted. All non-human reads from all KhoeSan samples subject to exome capture were used to generate this histogram.

Supplementary Material

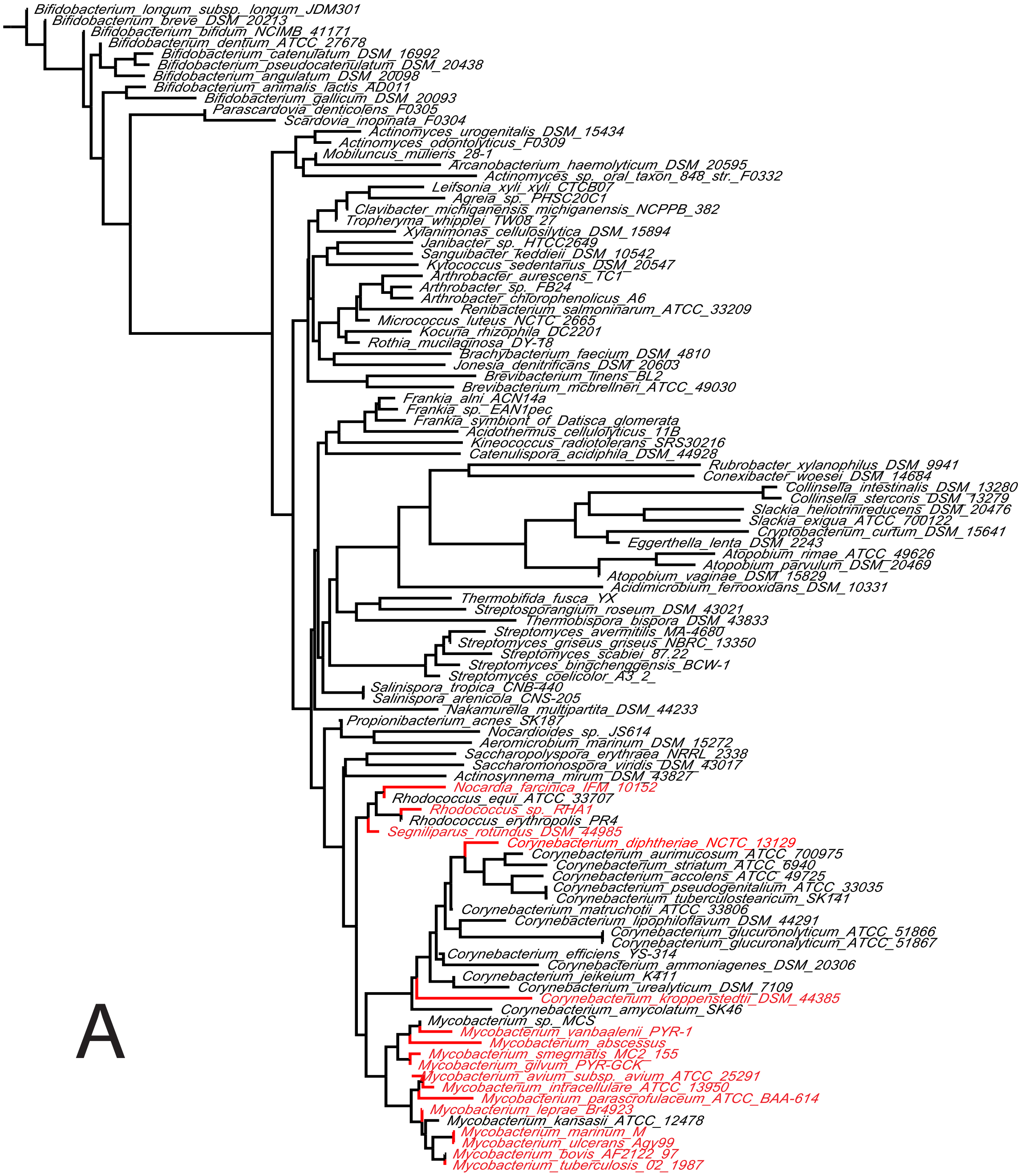

A

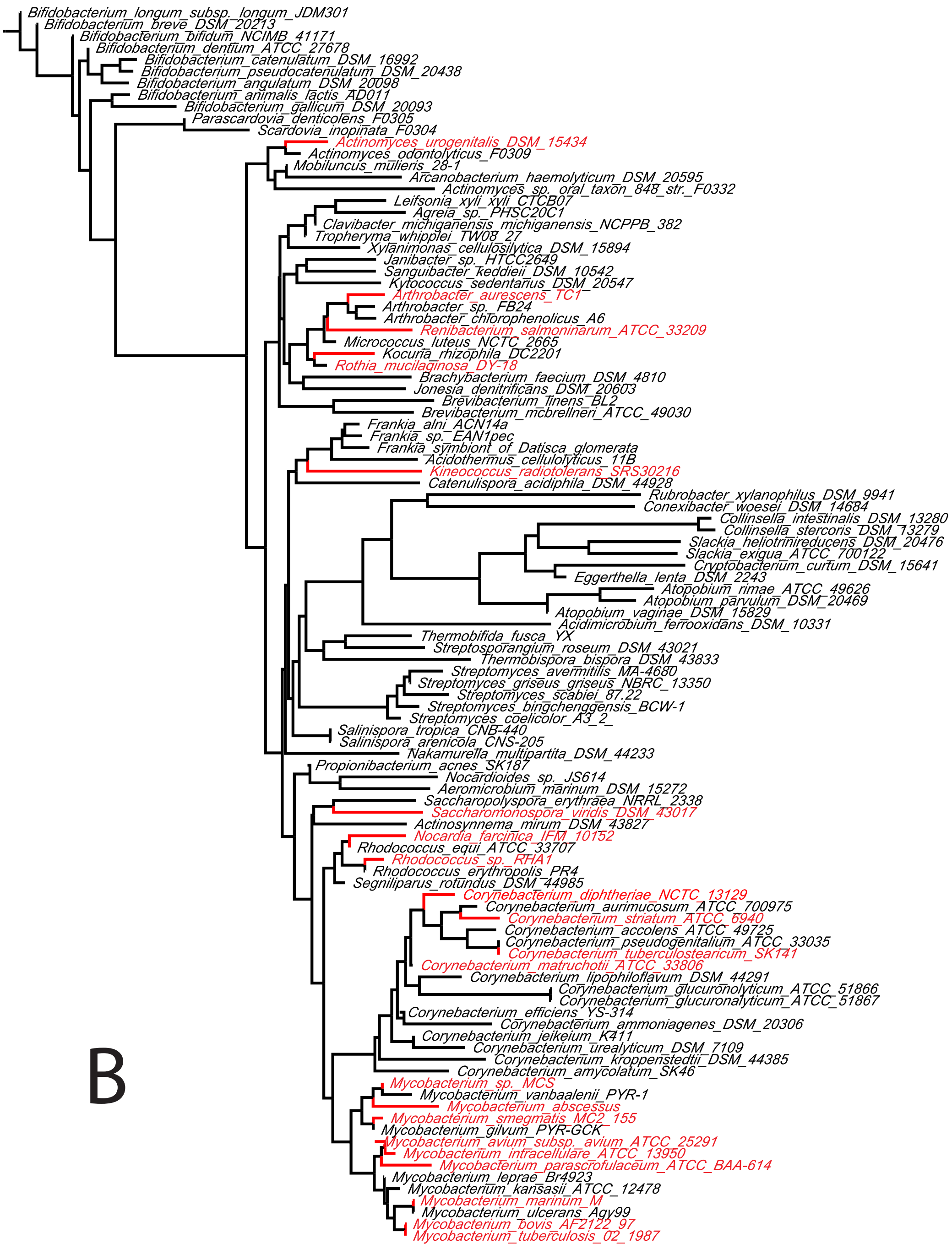
Bifidobacterium_longum_subsp._longum_JDM301
Bifidobacterium_breve_DSM_20213
Bifidobacterium_bifidum_NCIMB_41171
Bifidobacterium_dentium_ATCC_27678
Bifidobacterium_catenulatum_DSM_16992
Bifidobacterium_pseudocatenulatum_DSM_20438
Bifidobacterium_angulatum_DSM_20098
Bifidobacterium_animalis_lactis_AD011
Bifidobacterium_gallicum_DSM_20093
Parascardovia_denticolens_F0305
Scardovia_inopinata_F0304
Actinomyces_urogenitalis_DSM_15434
Actinomyces_odontolyticus_F0309
Mobiluncus_mulieris_28-1
Arcanobacterium_haemolyticum_DSM_20595
Actinomyces_sp._oral_taxon_848_str._F0332
Leifsonia_xyli_xyli_CTCB07
Agreia_sp._PHSC20C1
Clavibacter_michiganensis_michiganensis_NCPPB_382
Tropheryma_whipplei_TW08_27
Xylanimonas_cellulosilytica_DSM_15894
Janibacter_sp._HTCC2649
Sanguibacter_keddieii_DSM_10542
Kytococcus_sedentarius_DSM_20547
Arthrobacter_aurescens_TC1
Arthrobacter_sp._FB24
Arthrobacter_chlorophenolicus_A6
Renibacterium_salmoninarum_ATCC_33209
Micrococcus_luteus_NCTC_2665
Kocuria_rhizophila_DC2201
Rothia_mucilaginosa_DY-18
Brachybacterium_faecium_DSM_4810
Jonesia_denitrificans_DSM_20603
Brevibacterium_linens_BL2
Brevibacterium_mcbrellneri_ATCC_49030
Frankia_alni_ACN14a
Frankia_sp._EAN1pec
Frankia_symbiont_of_Datisca_glomerata
Acidothermus_cellulolyticus_11B
Kineococcus_radiotolerans_SRS30216
Catenulispora_acidiphila_DSM_44928
Rubrobacter_xylanophilus_DSM_9941
Conexibacter_woesei_DSM_14684
Collinsella_intestinalis_DSM_13280
Collinsella_stercoris_DSM_13279
Slackia_heliotrinireducens_DSM_20476
Slackia_exigua_ATCC_700122
Cryptobacterium_curtum_DSM_15641
Eggerthella_lenta_DSM_2243
Atopobium_rimae_ATCC_49626
Atopobium_parvulum_DSM_20469
Atopobium_vaginae_DSM_15829
Acidimicrobium_ferrooxidans_DSM_10331
Thermobifida_fusca_YX
Streptosporangium_roseum_DSM_43021
Thermobispora_bispora_DSM_43833
Streptomyces_avermitilis_MA-4680
Streptomyces_griseus_griseus_NBRC_13350
Streptomyces_scabiei_87.22
Streptomyces_bingchenggensis_BCW-1
Streptomyces_coelicolor_A3_2_
Salinispora_tropica_CNB-440
Salinispora_arenicola_CNS-205
Nakamurella_multipartita_DSM_44233
Propionibacterium_acnes_SK187
Nocardioides_sp._JS614
Aeromicrobium_marinum_DSM_15272
Saccharopolyspora_erythraea_NRRL_2338
Saccharomonospora_viridis_DSM_43017
Actinosynnema_mirum_DSM_43827
Nocardia_farcinica_IFM_10152
Rhodococcus_equi_ATCC_33707
Rhodococcus_sp._RHA1
Rhodococcus_erythropolis_PR4
Segniliparus_rotundus_DSM_44985
Corynebacterium_diphtheriae_NCTC_13129
Corynebacterium_aurimucosum_ATCC_700975
Corynebacterium_striatum_ATCC_6940
Corynebacterium_accolens_ATCC_49725
Corynebacterium_pseudogenitalium_ATCC_33035
Corynebacterium_tuberculostearicum_SK141
Corynebacterium_matruchotii_ATCC_33806
Corynebacterium_lipophiloflavum_DSM_44291
Corynebacterium_glucuronolyticum_ATCC_51866
Corynebacterium_glucuronalyticum_ATCC_51867
Corynebacterium_efficiens_YS-314
Corynebacterium_ammoniagenes_DSM_20306
Corynebacterium_jeikeium_K411
Corynebacterium_urealyticum_DSM_7109
Corynebacterium_kroppenstedtii_DSM_44385
Corynebacterium_amycolatum_SK46
Mycobacterium_sp._MCS
Mycobacterium_vanbaalenii_PYR-1
Mycobacterium_abscessus
Mycobacterium_smegmatis_MC2_155
Mycobacterium_gilvum_PYR-GCK
Mycobacterium_avium_subsp._avium_ATCC_25291
Mycobacterium_intracellulare_ATCC_13950
Mycobacterium_parascrofulaceum_ATCC_BAA-614
Mycobacterium_leprae_Br4923
Mycobacterium_kansasii_ATCC_12478
Mycobacterium_marinum_M
Mycobacterium_ulcerans_Agy99
Mycobacterium_bovis_AF2122_97
Mycobacterium_tuberculosis_02_1987
B

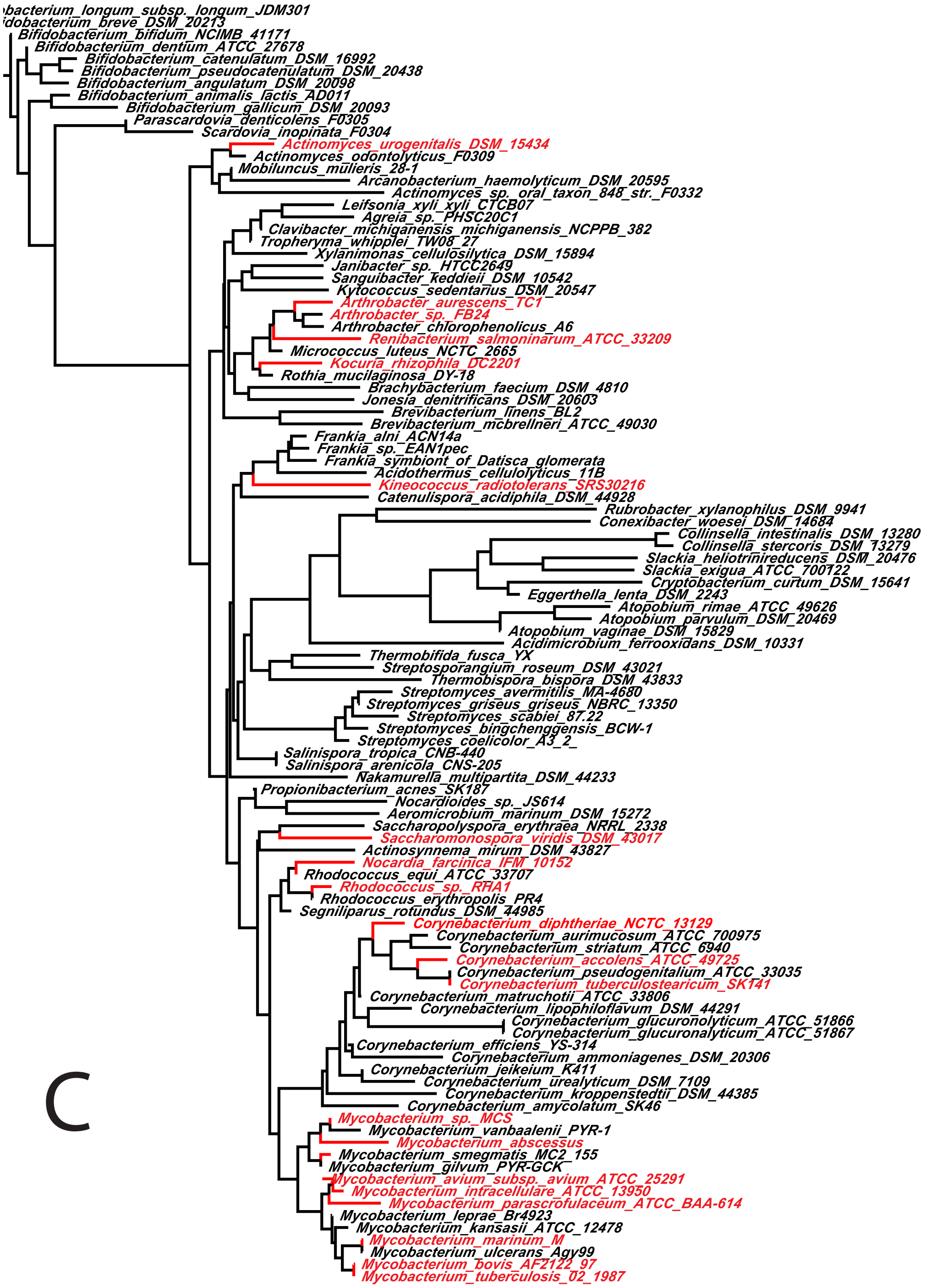

Figure S8 *The phylogenetic distribution of three non-human exome capture sequences that map with high fidelity to Mycobacterium tuberculosis*. Each read's distribution is illustrated in the SSU-rRNA (i.e., 16S rRNA) phylogenies above (A, B, and C), which were generated by the Joint Genomes Institutes via the IMG database using PHYLIP. Lineages colored in red represent species whose genome recruit the read with equal fidelity as the *M. tuberculosis* genome. Lineages colored in black represent reference genomes that did not recruit the read. Only Actinobacteria are included in these phylogenies.

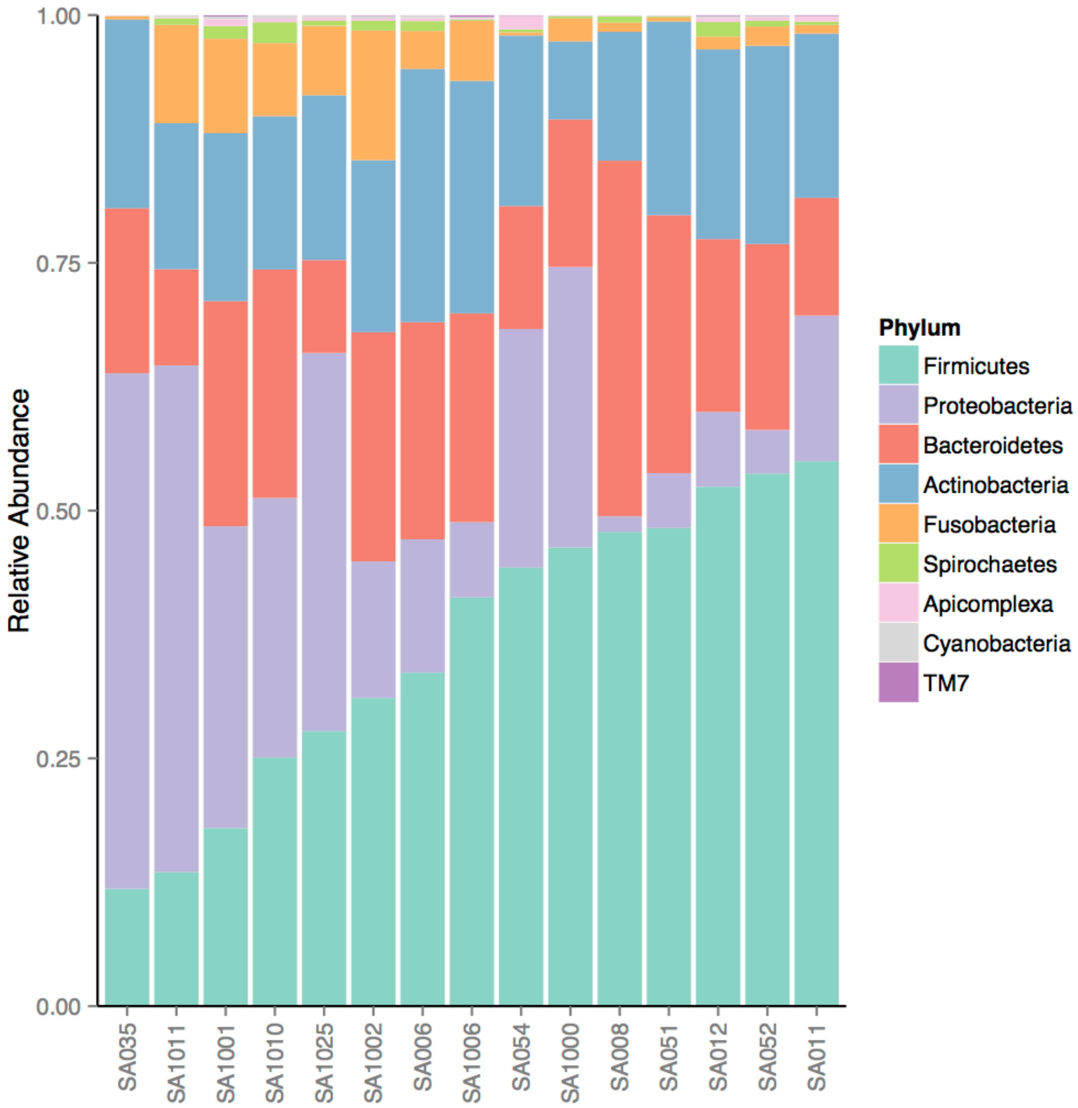

Figure S9 *The phylum-level structure of the oral microbiome structure varies among the KhoeSan*. Each of the above stacked bar plots illustrates the relative abundance (y-axis) of the most abundant oral phyla for each of the 15 KhoeSan individuals (x-axis). Relative abundance was measured as the fraction of high-quality reads that were recruited to a microbial genome of a particular taxonomic rank using conservative recruitment settings (Methods).
